# Epidemiological modeling of COVID-19 data with Advanced statistical inference based on Type-II progressive censoring

**DOI:** 10.1016/j.heliyon.2024.e36774

**Published:** 2024-08-30

**Authors:** Naif Alotaibi, A.S. Al-Moisheer, Amal S. Hassan, Ibrahim Elbatal, Salem A. Alyami, Ehab M. Almetwally

**Affiliations:** a Department of Mathematics and Statistics, Faculty of Science, Imam Mohammad Ibn Saud Islamic University (IMSIU), Riyadh, 11432, Saudi Arabia; b Department of Mathematics, College of Science, Jouf University, P.O. Box 848, Sakaka, 72351, Saudi Arabia; c Faculty of Graduate Studies for Statistical Research, Cairo University, 12613, Giza, Egypt; d Faculty of Business Administration, Delta University for Science and Technology, Gamasa, 11152, Egypt

**Keywords:** Kavya-Manoharan, Progressive type-II censoring, Statistical inference, Bayes prediction, COVID-19 datasets, 62F15, 62N01, 62N05

## Abstract

This research proposes the Kavya-Manoharan Unit Exponentiated Half Logistic (KM-UEHL) distribution as a novel tool for epidemiological modeling of COVID-19 data. Specifically designed to analyze data constrained to the unit interval, the KM-UEHL distribution builds upon the unit exponentiated half logistic model, making it suitable for various data from COVID-19. The paper emphasizes the KM-UEHL distribution's adaptability by examining its density and hazard rate functions. Its effectiveness is demonstrated in handling the diverse nature of COVID-19 data through these functions. Key characteristics like moments, quantile functions, stress-strength reliability, and entropy measures are also comprehensively investigated. Furthermore, the KM-UEHL distribution is employed for forecasting future COVID-19 data under a progressive Type-II censoring scheme, which acknowledges the time-dependent nature of data collection during outbreaks. The paper presents various methods for constructing prediction intervals for future-order statistics, including maximum likelihood estimation, Bayesian inference (both point and interval estimates), and upper-order statistics approaches. The Metropolis-Hastings and Gibbs sampling procedures are combined to create the Markov chain Monte Carlo simulations because it is mathematically difficult to acquire closed-form solutions for the posterior density function in the Bayesian framework. The theoretical developments are validated with numerical simulations, and the practical applicability of the KM-UEHL distribution is showcased using real-world COVID-19 datasets.

## 1 Introduction

For the interpretation of real-world events, a notable feature of the majority of the novel distributions is that they are formulated using special functions or additional parameters based on either the full real line or the positive real line. Recent variety models offer great possibilities for fitting complex and asymmetric random events and solving real-world challenges. As a result, numerous models have been created and researched in the literature. On the other hand, using unit distributions is essential for modeling proportions that are frequently seen in business, medical applications, and risk analysis, to name a few. The beta distribution, which is a practical and effective model in many fields of statistics, is the most well-known unit distribution in the statistical literature. Unfortunately, the data may not be sufficiently explained by the data model due to limitations. For addressing bounded data sets in various fields, a number of probability distributions were suggested in this regard. Among them are the Johnson SB distribution [1], the Topp-Leone distribution [2], the unit gamma distribution [3,4], Kumaraswamy distribution [5], unit-Birnbaum-Saunders distribution [6], unit-Weibull distribution [7,8], unit power Burr X distribution [9], unit-Gompertz distribution [10], unit-inverse Gaussian distribution [11], unit-Burr-XII distribution [12], unit-Gamma/Gompertz distribution [13], unit exponentiated Lomax distribution [14], unit–exponentiated half-logistic distribution [15], unit power Lomax distribution [16], and unit inverse exponentiated Weibull distribution [17], among others. Specifically, the unit exponentiated half logistic (UEHL) distribution is of intreset here, with the probability density function (PDF) and cumulative distribution function (CDF) as follows:

(1) $$g\left(t\right)=\frac{2\kappa \varsigma t^{\kappa -1}}{{\left(1+t^{\kappa }\right)}^{2}}{\left(\frac{1-t^{\kappa }}{1+t^{\kappa }}\right)}^{\varsigma -1},t\in \left(0,1\right)\text{,}$$

and,

(2) $$G\left(t\right)=1-{\left(\frac{1-t^{\kappa }}{1+t^{\kappa }}\right)}^{\varsigma },t\in \left(0,1\right)\text{,}$$

where, ς>0, and κ>0 are the shape parameters. The UEHL distribution has different forms of asymmetric shapes, such as right-skewed, left-skewed, reverse-J, and U-shaped. A compound family based on the UEHL distribution with a power series distribution was considered by Ref. [18]. One of the advantages of the UEHL distribution is that it doesn't contain any special functions. This distribution is incredibly adaptable and provides a strong basis for bounded data statistical modeling and analysis. The distribution has a wide range of practical applications; it has been used in fields such as material strength, economic development, and medical statistics, most notably in COVID-19 data analysis, demonstrating its usefulness in realistic situations (see Ref. [15]). One might consider the two-parameter UEHL distribution with domain (0,1) as an alternative for the following distributions: Kumaraswamy, beta, unit Weibull, Marshall–Olkin–Kumaraswamy, Kumaraswamy–Kumaraswamy unit Burr–XII, and unit generalized log Burr XII.

In many domains, mathematical models are tremendously significant. They were created with the intention of combating the pandemic. Epidemiological models are models used to combat different types of epidemics. In the absence of a suitable vaccine or targeted antivirals, mathematical modeling is essential for improving understanding of disease dynamics and for developing strategies to control the rapid spread of illnesses. Researchers have put forth a number of mathematical models to assess the dynamic behavior and transmission of certain diseases, which might help with illness control or even future event prediction [[19], [20], [21], [22], [23], [24]]. Midway through March 2020, the World Health Organization declared the novel coronavirus illness (COVID-19) to be a worldwide pandemic. This disease is brought on by an infection with the SARS-CoV-2 virus. The emerging strains of the coronavirus pandemic pose a serious threat to humankind. Globalization has made it easier for illnesses to spread quickly across small distances. This has an impact on the public health care system and impedes the emerging and impoverished nations' ability to prosper economically. In recent times, several mathematical models have been examined to comprehend the intricate dynamics of the newly discovered COVID-19 [[25], [26], [27], [28], [29], [30]]. Another crucial element in restricting the spread of diseases is the mathematical modeling of infectious diseases and the impact of media [[31], [32], [33], [34]].

Modeling actual events using probability distributions is one of statistics' most crucial responsibilities. Many applied sciences, including medicine, engineering, and finance, among others, rely heavily on modeling arious real data sets. The created family of distributions greatly influences the effectiveness of statistical analysis techniques; hence, new statistical models have undergone extensive development. The method of expanding a family of distributions by adding new parameters is acknowledged in the statistical literature [[35], [36], [37], [38], [39], [40], [41], [42]]. Recently, the Dinesh-Umesh-Sanjay (DUS) transformation approach was introduced by Ref. [43] to obtain novel lifetime distributions. Creating new classes of parsimonious distributions with no extra parameters is the main goal of this transformation. The DUS transformation produces a new CDF written as:

$$W\left(t\right)=\frac{e^{G\left(t\right)}-1}{e-1};\;t\in R\text{,}$$

where *G*(.) is the CDF of the parent distribution. A generalized DUS transformation was just recently put forth by Ref. [44] to generate some interesting lifetime distributions. In order to create new parsimonious families of distributions, Ref. [45] created a parsimonious transformation, called the Kavya–Manoharan (KM) family of distributions. The CDF and PDF of the KM transformation, for t∈R, are defined as:

(3) $$W\left(t\right)=e^{*}\left[1-e^{-G\left(t;\Theta \right)}\right]\text{,}$$

(4) $$w\left(t\right)=e^{*}g\left(t;\Theta \right)e^{-G\left(t;\Theta \right)}\text{,}$$

where $e^{*}=\frac{e}{e-1}\text{,}$ *G*(.) is the CDF, and *g*(.) is the PDF of the base-line distributions and Θ is the set of parameters. Reference [45] introduced two new models by using exponential as well as Weibull distributions as baseline distributions. Using the KM transformations, Ref. [46] proposed the bivariate KM exponentiated-Weibull distribution in step stress accelerated life tests. For more studies, the reader can refer to Refs. [[47], [48], [49], [50]].

Due to time constraints and the high expense of conducting the experiment, censored samples are typically used in life-testing experiments when the experimenter wants to end the study before all units have failed. The two primary categories of censoring techniques are Type-I and Type-II. The fundamental drawback of Type I and Type II- censoring sample (TII-CS) techniques is that they do not permit the removal of units from an experiment at any point other than the termination point. One key technique for gathering information in these lifetime studies is progressive TII-CS (PTII-CS). The PTII-CS method is a broader censoring scheme in which the surviving units can be eliminated during the experimentation. In this scheme, *m* (*m* < *n*) failures are thoroughly seen once *n* units are placed on a life-testing experiment at time zero. Suppose that *R*_*i*_ represents the number of units removed at the time of the ith failure. The *R*_1_ number of surviving units is eliminated at the first failure time *T*_1:m:n_ from the experiment at random. The remaining *R*_2_ units are then randomly removed from the experiment after the second failure time *T*_2:m:n_.. This process continues until all of the $R_{m}=n-m-R_{1}-R_{2}-...-R_{m-1}$ surviving units are taken out of the experiment at the time of the mth failure, *T*_m:m:n_. Prior to the life testing experiment, *m* and (*R*_1_, *R*_2_, …, *R*_*m*_) are fixed in this case. Obtaining inferences about the unknown characteristics of the lifetime distribution under investigation is one of the primary goals of reliability and life testing experiments [51]. In many domains, including the medical and engineering sciences, prediction based on censored data is a crucial topic. Predicting the type of a future sample based on a current sample is one of a life-testing experiment's key goals. In the context of quality and reliability analysis, the problem of mean, smallest, and largest observation prediction in future sample is one of interest and importance. In this regard, researchers are interested in estimating unknown parameters and/or drawing conclusions from censored (future) observations.

The major goal of this paper is to introduce a more flexible UEHL distribution that is based on the KM transformation. The model that has been proposed is referred to as the Kavya–Manoharan UEHL (KM-UEHL) distribution. The following factors led us to choose the recommended model for further investigation: Here are the specifics.

⁃ To improve the versatility of the traditional UEHL distribution in simulating different occurrences. The KM-UEHL model displays rising, decreasing, J-shaped, and U-shaped hazard rates. Therefore, the KM-UEHL model is useful in situations when the UEHL model is not realistically relevant.
⁃ The analytical moments expression, probability weighted moments (PWM), quantile function (QF), uncertainty measures, stress-strength (S-S) reliability, moments of residual and reversed failure rate, and entropy measures are some of the major statistical features that are derived for the KM-UEHL distribution.
⁃ To estimate the involved parameters in the KM-UEHL distribution using maximum likelihood and Bayesian techniques based on PTII-CS. The Bayesian estimate (BE) of the KM-UEHL distribution's model parameters using gamma prior is determined under the symmetric loss function. The approximate confidence interval (ACI) estimates of the model parameters and the highest posterior density (HPD) interval estimates are obtained.
⁃ The predictive interval of unobserved units in the same sample is created (one sample prediction), as is the predictive interval for the subsequent sample based on the present sample (two-sample prediction).
⁃ Markov chain Monte Carlo (MCMC) methods are used to approximate the BEs and create the HPD intervals because the BEs cannot be derived in closed-form. To evaluate the effectiveness of the suggested approaches using various options of effective sample size, a Monte Carlo simulation analysis is carried out.
⁃ The effectiveness of the proposed model is demonstrated by its superior performance compared to other established models, as illustrated through two real-world datasets.

The following describes the scenario for this essay: The KM-UEHL distribution's structure is explained in Section 2. Section 3 presents the major characteristics of the KM-UEHL distribution. Section 4 considers the maximum likelihood (ML) and Bayesian estimation techniques for estimating the unknown parameters. The Bayesian prediction issue of the unidentified observations from the censored sample is introduced in Section 5. In Section 6, a Monte Carlo simulation using numerical comparisons is carried out. The application of the novel distribution to an actual data set is covered in Section 7 of the paper. The paper ends in Section 8.

## 2 Formation of the KM-UEHL distribution

If the PDF given in Equation (1) and CDF given in Equation (2) are inserted in Equation (3) and Equation (4), then a random variable *T* is said to have the KM-UEHL distribution. A random variable *T* has the PDF and CDF, for $\varpi =\left(\varsigma ,\kappa \right)\in R^{+}\text{,}$ which are determined by:

(5) $$W\left(t\right)=e^{*}\left[1-e^{-\left[1-H\left(t,\varpi \right)\right]}\right];\;0<t<1\text{,}$$

and,

(6) $$w\left(t\right)=\frac{2e^{*}\kappa \varsigma t^{\kappa -1}{\left(1-t^{\kappa }\right)}^{\varsigma -1}}{{\left(1+t^{\kappa }\right)}^{\varsigma +1}}e^{-\left[1-H\left(t,\varpi \right)\right]};\;0<t<1\text{.}$$

where, $H\left(t,\varpi \right)={\left(\frac{1-t^{\kappa }}{1+t^{\kappa }}\right)}^{\varsigma }$. The reliability function and hazard function (HF) of *T* are determined via:

$$\bar{W}\left(t\right)=1-e^{*}\left[1-e^{-\left[1-H\left(t,\varpi \right)\right]}\right]\text{,}$$

and,

$$\tau \left(t\right)=\frac{2\kappa \varsigma t^{\kappa -1}{\left(1-t^{\kappa }\right)}^{\varsigma -1}e^{-\left[1-H\left(t,\varpi \right)\right]}}{{\left(1+t^{\kappa }\right)}^{\varsigma +1}\left\{e-1-e\left[1-e^{-\left[1-H\left(t,\varpi \right)\right]}\right]\right\}}\text{.}$$

The asymptotes of w(t) and τ(t) at *t* → 0 and *t* → 1 are studied below:

$$\lim_{t\to 0}w\left(t\right)=\lim_{t\to 0}\tau \left(t\right)=\left\{ \begin{array}{cc} \infty & \kappa <1\\ \frac{2e\tau }{\left(e-1\right)} & \kappa =1\\ 0 & \kappa >1 \end{array}\right.,\lim_{t\to 1}w\left(t\right)=\lim_{t\to 1}\tau \left(t\right)=\left\{ \begin{array}{cc} \infty & \tau <1\\ \frac{\kappa }{2^{\tau }\left(e-1\right)} & \tau =1\\ 0 & \tau >1 \end{array}\right.\text{.}$$

**Fig. 1:**
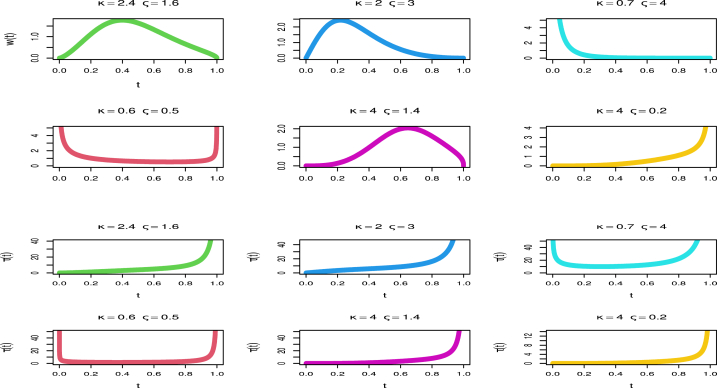
A graphical exploration of KM-UEHL distribution shapes.

Fig. 1 (top) demonstrates the possibility of a unimodal, left-skewed (ς=1.4,κ=4), decresing-shaped (ς=4,κ=0.7), U-shaped (ς=0.5,κ=0.6), and right-skewed (ς=0.2,κ=4) PDF for the KM-UEHL distribution. Fig. 1 demonstrates that the HF of the KM-UEHL distribution includes U-shaped, J-shaped, increasing, constant, and decreasing forms.

## 3 Statistical properties

A few mathematical characteristics of the KM-UEHL distribution are listed in this section.

### 3.1 Probability weighted moments

The class of PWMs is used to estimate distribution parameters and quantiles. The PWM of the KM-UEHL distribution, for which *u* and *v* are positive integers, is specified by:

(7) $$\gamma_{u,v}=E\left[T^{u}W{\left(t\right)}^{v}\right]=\int_{-\infty }^{\infty }t^{u}w\left(t\right){\left(W\left(t\right)\right)}^{v}dt\text{.}$$

The PWM of the KM-UEHL distribution is determined as follows by setting Equations (5), (6) in Equation (7):

(8) $$\gamma_{u,v}=2\kappa \varsigma {\left(e^{*}\right)}^{v+1}\int_{0}^{1}e^{-\left[1-H\left(t,\varpi \right)\right]}\frac{t^{u+\kappa -1}{\left(1-t^{\kappa }\right)}^{\varsigma -1}}{{\left(1+t^{\kappa }\right)}^{\varsigma +1}}{\left[1-e^{-\left[1-H\left(t,\varpi \right)\right]}\right]}^{v}dt\text{.}$$

Using the binomial and exponential expansions in the last term in Equation (8), then

$$\gamma_{u,v}=\sum_{c_{2},c_{4}=0}^{\infty }\omega_{c_{1},c_{2},c_{3},c_{4}}\left(\varsigma \right)k\int_{0}^{1}{\left(1-t^{\kappa }\right)}^{\varsigma \left(c_{3}+1\right)-1}t^{u+\kappa +\kappa c_{4}-1}dt\text{,}$$

where$\omega_{c_{1},c_{2},c_{3},c_{4}}\left(\varsigma \right)=\sum_{c_{1}=0}^{v}\sum_{c_{3}=0}^{c_{2}}\frac{{\left(-1\right)}^{c_{1}+c_{2}+c_{3}}{\left(c_{1}+1\right)}^{c_{2}}2\varsigma }{c_{2}!}\left(\begin{array}{c} v\\ c_{1} \end{array}\right)\left(\begin{array}{c} c_{2}\\ c_{3} \end{array}\right)\left(\begin{array}{c} \varsigma \left(c_{3}+1\right)+c_{4}\\ c_{4} \end{array}\right){\left(e^{*}\right)}^{v+1}\text{.}$

As a result, after simplification, the PWM of the KM-UEHL distribution has the following structure:

$$\gamma_{u,v}=\sum_{c_{2},c_{4}=0}^{\infty }\omega_{c_{1},c_{2},c_{3},c_{4}}\left(\varsigma \right)Β\left(\frac{u}{\kappa }+c_{4}+1,\varsigma \left(c_{3}+1\right)\right)\text{,}$$

where B(.,.) is the beta function (BFu).

### 3.2 Some moments measures

The nth moment of *T* is given by $\mu_{n}^{\prime }=\int_{-\infty }^{\infty }t^{n}w\left(t\right)dt\text{.}$ Using PDF presented in Equation (6), then

(9) $$\mu_{n}^{\prime }=2e^{*}\varsigma \;\kappa \int_{0}^{1}\frac{\varsigma t^{n+\kappa -1}{\left(1-t^{\kappa }\right)}^{\varsigma -1}}{{\left(1+t^{\kappa }\right)}^{\varsigma +1}}e^{-[1-H\left(t,\varpi \right)]}dt\text{.}$$

Applying, the binomial and exponential expansions in Equation (9), provides

$$\mu_{n}^{\prime }=2e^{*}\varsigma \;\kappa \sum_{c_{1}=0}^{\infty }\sum_{c_{2}=0}^{c_{1}}\left(\begin{array}{c} c_{1}\\ c_{2} \end{array}\right)\frac{{\left(-1\right)}^{c_{1}+c_{2}}}{c_{1}!}\int_{0}^{1}\frac{t^{n+\kappa -1}{\left(1-t^{\kappa }\right)}^{\varsigma \left(c_{2}+1\right)-1}}{{\left(1+t^{\kappa }\right)}^{\varsigma \left(c_{2}+1\right)+1}}dt=\sum_{c_{1},c_{3}=0}^{\infty }\varphi_{c_{1},c_{2},c_{3}}\left(\varsigma \right)\kappa \int_{0}^{1}t^{n+\kappa c_{3}+\kappa -1}{\left(1-t^{\kappa }\right)}^{\varsigma \left(c_{2}+1\right)-1}dt\text{.}$$

where $\varphi_{c_{1},c_{2},c_{3}}\left(\varsigma \right)=\sum_{c_{2}=0}^{c_{1}}\left(\begin{array}{c} c_{1}\\ c_{2} \end{array}\right)\left(\begin{array}{c} \varsigma \left(c_{2}+1\right)+c_{3}\\ c_{3} \end{array}\right)\frac{{\left(-1\right)}^{c_{1}+c_{2}+c_{3}}}{c_{1}!}e^{*}\varsigma \text{.}$

After being simplified, the *n*th moment of the KM-UEHL distribution has the following formula:

(10) $$\mu_{n}^{\prime }=\sum_{c_{1},c_{3}=0}^{\infty }\phi_{c_{1},c_{2},c_{3}}\left(\varsigma \right)Β\left(\frac{n}{\kappa }+c_{3}+1,\varsigma \left(c_{2}+1\right)\right)\text{.}$$

By substituting *n* = 1, 2, 3, and 4 in Equation (10), one can derive the first four moments of the MK-UEHL distribution.

### 3.3 Residual life and reversed failure rate function

As shown below, the nth moment of the residual life (RL) of *T* is defined:

(11) $$\vartheta_{n}\left(z\right)=E\left(\left(T-z\right)|T>z\right)=\frac{1}{\bar{W}\left(z\right)}\int_{z}^{\infty }{\left(t-z\right)}^{n}dW\left(t\right)\text{.}$$

Using the binomial expansion for ${\left(t-z\right)}^{n}$ in Equation (11) and utilizing Equation (6) results in the following:

(12) $$\vartheta_{n}\left(z\right)=\frac{2e^{*}\varsigma \;\kappa }{\bar{W}\left(z\right)}\sum_{l_{1}=0}^{n}{\left(-1\right)}^{l_{1}}\left(\begin{array}{c} n\\ l_{1} \end{array}\right)z^{n-l_{1}}\int_{z}^{1}\frac{t^{l_{1}+\kappa -1}{\left(1-t^{\kappa }\right)}^{\varsigma -1}}{{\left(1+t^{\kappa }\right)}^{\varsigma +1}}e^{-\left[1-H\left(t,\varpi \right)\right]}dt\text{.}$$

Applying, the binomial and exponential expansions in Equation (12), provides

$$\vartheta_{n}\left(z\right)=\sum_{l_{2},l_{4}=0}^{\infty }\frac{\Delta_{l_{1},l_{2},l_{3},l_{4}}\kappa }{\bar{W}\left(z\right)}\int_{z}^{1}t^{l_{1}+\kappa +\kappa l_{4}-1}{\left(1-t^{\kappa }\right)}^{\varsigma \left(l_{3}+1\right)-1}dt\text{,}$$

$$\Delta_{l_{1},l_{2},l_{3},l_{4}}\left(\varsigma \right)=\sum_{l_{1}=0}^{n}\sum_{l_{3}=0}^{l_{2}}\frac{{\left(-1\right)}^{l_{1}+l_{2}+l_{3}+l_{4}}e^{*}\varsigma \;z^{n-l_{1}}}{l_{2}!}\left(\begin{array}{c} n\\ l_{1} \end{array}\right)\left(\begin{array}{c} \varsigma \left(l_{3}+1\right)+l_{4}\\ l_{4} \end{array}\right)\left(\begin{array}{c} l_{2}\\ l_{3} \end{array}\right)$$

Let $y=1-t^{\kappa }\text{,}$ then $\vartheta_{n}\left(z\right)\text{,}$ is as follows:

(13) $$\vartheta_{n}\left(z\right)=\sum_{l_{2},l_{4}=0}^{\infty }\frac{\Delta_{l_{1},l_{2},l_{3},l_{4}}\left(\varsigma \right)}{\bar{W}\left(z\right)}Β\left(\frac{l_{1}}{\kappa }+l_{4}+1,\varsigma \left(l_{3}+1\right),1-z^{\kappa }\right)\text{,}$$

where, B(.,.,*x*) is the incomplete BFu. The mean RL or the life expectation at age *z* is determined by setting *n* = 1 in Equation (13).

Next, the *nt*h moment of the reversed residual (RR) life of *T* is then:

(14) $${\overset{¨¨}{\vartheta }}_{n}\left(t\right)=E\left(\left(T-z\right)|T<z\right)=\frac{1}{W\left(z\right)}\int_{0}^{z}{\left(z-t\right)}^{n}dW\left(t\right)\text{.}$$

Using the binomial expansion for ${\left(t-z\right)}^{n}\text{,}$ then using binomial and exponential expansions in Equation (14), provide the following:

(15) $${\overset{¨¨}{\vartheta }}_{n}\left(z\right)=\sum_{l_{2},l_{4}=0}^{\infty }\frac{\Delta_{l_{1},l_{2},l_{3},l_{4}}\left(\varsigma \right)z^{n-l_{1}}\kappa }{W\left(z\right)}\int_{0}^{z}t^{l_{1}+\kappa +kl_{4}-1}{\left(1-t^{\kappa }\right)}^{\varsigma \left(l_{3}+1\right)-1}dt=\sum_{l_{2},l_{4}=0}^{\infty }\frac{\Delta_{l_{1},l_{2},l_{3},l_{4}}\left(\varsigma \right)z^{n-l_{1}}}{W\left(z\right)}Β\left(\frac{l_{1}}{\kappa }+l_{4}+1,\varsigma \left(l_{3}+1\right),z^{\kappa }\right)\text{.}$$

The mean inactivity time, also known as the mean waiting time is determined, for *n* = 1 in Equation (15).

### 3.4 Stress-strength parameter

Assuming *T*_1_ and *T*_2_ be two independent random variables, where *T*_1_∼ KM-UEHL ($\kappa ,\varsigma_{1}$) and *T*_2_∼KM-UEHL $\left(\kappa ,\varsigma_{2}\right)\text{,}$ then the S-S, say $S^{•}$ is defined as:

(16) $$S^{•}=\int_{-\infty }^{\infty }w_{1}\left(t\right)W_{2}\left(t\right)dt\text{.}$$

The S-S parameter can be determined by substituting Equations (5), (6) into Equation (16), as shown below

(17) $$S^{•}=e^{*}\int_{0}^{1}\frac{2\kappa e^{*}\varsigma_{1}t^{\kappa -1}{\left(1-t^{\kappa }\right)}^{\varsigma_{1}-1}}{{\left(1+t^{\kappa }\right)}^{\varsigma_{1}+1}}\left[1-e^{-\left[1-H\left(t,\varpi_{2}\right)\right]}\right]e^{-\left[1-H\left(t,\varpi_{1}\right)\right]}dt\text{,}$$

where, $\varpi_{1}=\left(\kappa ,\varsigma_{1}\right)$ and $\varpi_{2}=\left(\kappa ,\varsigma_{2}\right)\text{.}$ Using exponential expansion in Equation (17)

(18) $$S^{•}=e^{*}\left[1-\sum_{c_{1},c_{2}=0}^{\infty }\frac{{\left(-1\right)}^{c_{1}+c_{2}}2e^{*}\kappa \varsigma_{1}}{c_{1}!c_{2}!}\int_{0}^{1}\frac{t^{\kappa -1}{\left(1-t^{\kappa }\right)}^{\varsigma_{1}-1}}{{\left(1+t^{\kappa }\right)}^{\varsigma_{1}+1}}{\left[1-H\left(t,\varpi_{1}\right)\right]}^{c_{1}}{\left[1-H\left(\varpi_{2}\right)\right]}^{c_{2}}dt\right]\text{.}$$

Again, using binomial expansions in Equation (18)

$$S^{•}=e^{*}\left[1-\sum_{c_{1},c_{2}=0}^{\infty }\delta_{c_{1},c_{2},c_{3},c_{4}}\left(\varsigma_{1}\right)\kappa \int_{0}^{1}\frac{t^{\kappa -1}{\left(1-t^{\kappa }\right)}^{\varsigma_{1}+\varsigma_{1}c_{3}+\varsigma_{2}c_{4}-1}}{{\left(1+t^{\kappa }\right)}^{\varsigma_{1}+\varsigma_{1}c_{3}+\varsigma_{2}c_{4}+1}}dt\right]\text{,}$$

where $\delta_{c_{1},c_{2},c_{3},c_{4}}\left(\varsigma_{1}\right)=\sum_{c_{3}=0}^{c_{1}}\sum_{c_{4}=0}^{c_{2}}\left(\begin{array}{c} c_{1}\\ c_{3} \end{array}\right)\left(\begin{array}{c} c_{2}\\ c_{4} \end{array}\right)\frac{{\left(-1\right)}^{c_{1}+c_{2}+c_{3}+c_{4}}2e^{*}\varsigma_{1}}{c_{1}!c_{2}!}\text{.}$

Next, by applying binomial expansion to the above equation, gives

$$S^{•}=e^{*}\left[1-\sum_{c_{1}=0}^{\infty }\sum_{c_{2}=0}^{c_{1}}\delta_{c_{1},c_{2},c_{3},c_{4},c_{5}}^{•}\left(\varsigma_{1}\right)Β\left(c_{5}+1,\varsigma_{1}\left(c_{3}+1\right)+\varsigma_{2}c_{4}\right)\right]\text{,}$$

where$\delta_{c_{1},c_{2},c_{3},c_{4},c_{5}}^{•}\left(\varsigma_{1}\right)=\sum_{c_{5}=0}^{\infty }{\left(-1\right)}^{c_{5}}\left(\begin{array}{c} \varsigma_{1}\left(c_{3}+1\right)+\varsigma_{2}c_{4}+c_{5}\\ c_{5} \end{array}\right)\delta_{c_{1},c_{2},c_{3},c_{4}}\left(\varsigma_{1}\right)\text{.}$

### 3.5 Quantile function

By inverting CDFgiven in Equation (5), the following provides the QF of the MK-UEHL distribution

(19) $$Q\left(p\right)=W^{-1}\left(t\right)={\left(\frac{1-m^{*}}{1+m^{*}}\right)}^{\frac{1}{\kappa }},m^{*}={\left[1+\ln\left(1+pe^{*}\right)\right]}^{\varsigma }\text{,}$$

where *p* is a uniform distribution between 0 and 1. Setting *p* = 0.5, 0.75, and 0.25 in Equation (19) will allow us to determine the median (Q(0.5)), upper (Q(0.75)), and lower (Q(0.25)) quantiles. Additionally, Bowley(BW)'s skewness ($\delta_{1}$) and Moor (MO)'s kurtosis ($\delta_{2}$) are offered via the quantiles.

$$\delta_{1}=\frac{Q\left(0.75\right)-2Q\left(0.5\right)+Q\left(0.25\right)}{Q\left(0.75\right)-Q\left(0.25\right)}\text{,}$$

and

$$\delta_{2}=\frac{Q\left(0.875\right)-2Q\left(0.625\right)-Q\left(0.375\right)+Q\left(0.125\right)}{Q\left(0.75\right)-Q\left(0.25\right)}\text{,}$$

**Fig. 2:**
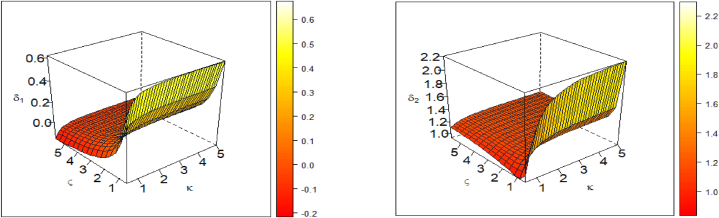
The 3D shapes of BW's skewness (left panel), and MO's kurtosis (right panel) of the KM-UEHL.

Fig. 2 illustrates the skewness and kurtosis of the KM-UEHL distribution in 3D plots. The left panel depicts the Bowley's skewness, while the right panel showcases the Moors' kurtosis.

Table 1 presents descriptive statistics, including minimum (Min), mean, median, variance (var.), maximum (Max.), $\delta_{1}$ and $\delta_{2}$ coefficients for various data sets. The minimum values, though not constant, are close or less to 0.1 initially but increase as the parameters increse. The mean values rise across the rows, while the var. Generally decreases, indicating that the data points become more concentrated around the mean. The var. Coefficients also show variations, suggesting that the spread of data around the mean narrows as the mean increases, leading to a tighter data distribution.

**Table 1:** Descriptive statistics with different parameters value of distribution.

ς	κ	Min.	Mean	Median	var.	Max.	$\delta_{1}$	$\delta_{2}$
0.5	0.5	0	0.3318	0.1976	0.1122	1	0.4221	1.0315
0.5	1.15	0	0.1377	0.0420	0.0425	0.9914	0.5843	1.7977
0.5	1.8	0	0.0733	0.0174	0.0175	0.9238	0.6070	2.0305
0.5	2.45	0	0.0448	0.0094	0.0081	0.7989	0.6163	2.1177
0.5	3.1	0	0.0299	0.0059	0.0042	0.6625	0.6173	2.1580
0.5	3.75	0	0.0213	0.0040	0.0023	0.5414	0.6189	2.1809
0.5	4.4	0	0.0159	0.0029	0.0014	0.4426	0.6212	2.1933
1.05	0.5	0.0004	0.4904	0.4620	0.1007	1	0.1052	0.9281
1.05	1.15	0.0002	0.2918	0.2209	0.0603	0.9959	0.2546	1.2169
1.05	1.8	0.0001	0.2061	0.1453	0.0367	0.9629	0.2778	1.2985
1.05	2.45	0.0001	0.1589	0.1086	0.0240	0.8986	0.2851	1.3288
1.05	3.1	0.0001	0.1292	0.0869	0.0168	0.8220	0.2885	1.3427
1.05	3.75	0.0001	0.1088	0.0725	0.0123	0.7466	0.2904	1.3504
1.05	4.4	0.0001	0.0940	0.0624	0.0094	0.6783	0.2904	1.3553
1.6	0.5	0.0065	0.5892	0.6025	0.0814	1	−0.0061	0.9684
1.6	1.15	0.0039	0.4082	0.3712	0.0598	0.9973	0.1295	1.1542
1.6	1.8	0.0029	0.3214	0.2820	0.0428	0.9755	0.1505	1.2070
1.6	2.45	0.0024	0.2696	0.2330	0.0322	0.9322	0.1573	1.2263
1.6	3.1	0.0021	0.2346	0.2013	0.0253	0.8793	0.1604	1.2352
1.6	3.75	0.0019	0.2093	0.1788	0.0205	0.8255	0.1620	1.2404
1.6	4.4	0.0017	0.1900	0.1618	0.0171	0.7751	0.1630	1.2432
2.15	0.5	0.0236	0.6563	0.6858	0.0652	1	−0.0613	1.0056
2.15	1.15	0.0160	0.4942	0.4783	0.0538	0.9980	0.0659	1.1483
2.15	1.8	0.0130	0.4121	0.3899	0.0421	0.9817	0.0858	1.1897
2.15	2.45	0.0113	0.3609	0.3382	0.0340	0.9491	0.0923	1.2049
2.15	3.1	0.0101	0.3252	0.3033	0.0284	0.9087	0.0952	1.2121
2.15	3.75	0.0093	0.2985	0.2777	0.0243	0.8670	0.0966	1.2155
2.15	4.4	0.0085	0.2776	0.2578	0.0213	0.8273	0.0978	1.2183
2.7	0.5	0.0506	0.7047	0.7406	0.0528	1	−0.0940	1.0339
2.7	1.15	0.0371	0.5593	0.5558	0.0469	0.9984	0.0278	1.1534
2.7	1.8	0.0314	0.4831	0.4723	0.0388	0.9854	0.0468	1.1886
2.7	2.45	0.0280	0.4343	0.4218	0.0328	0.9593	0.0531	1.2016
2.7	3.1	0.0258	0.3996	0.3867	0.0285	0.9266	0.0559	1.2077
2.7	3.75	0.0240	0.3731	0.3605	0.0252	0.8926	0.0572	1.2110
2.7	4.4	0.0226	0.3521	0.3398	0.0226	0.8599	0.0583	1.2128
3.25	0.5	0.0838	0.7412	0.7792	0.0434	1	−0.1157	1.0554
3.25	1.15	0.0648	0.6100	0.6139	0.0406	0.9987	0.0024	1.1602
3.25	1.8	0.0565	0.5396	0.5363	0.0349	0.9879	0.0209	1.1920
3.25	2.45	0.0514	0.4937	0.4881	0.0305	0.9660	0.0273	1.2033
3.25	3.1	0.0478	0.4605	0.4542	0.0271	0.9386	0.0298	1.2087
3.25	3.75	0.0451	0.4350	0.4284	0.0244	0.9099	0.0311	1.2115
3.25	4.4	0.0429	0.4146	0.4079	0.0224	0.8822	0.0319	1.2134
3.8	0.5	0.1200	0.7697	0.8079	0.0362	1	−0.1311	1.0719
3.8	1.15	0.0964	0.6505	0.6588	0.0351	0.9989	−0.0155	1.1670
3.8	1.8	0.0856	0.5853	0.5869	0.0311	0.9896	0.0025	1.1960
3.8	2.45	0.0790	0.5423	0.5415	0.0277	0.9709	0.0085	1.2067
3.8	3.1	0.0742	0.5109	0.5092	0.0251	0.9473	0.0110	1.2117
3.8	3.75	0.0706	0.4866	0.4844	0.0230	0.9224	0.0122	1.2143
3.8	4.4	0.0677	0.4669	0.4645	0.0213	0.8983	0.0133	1.2158

### 3.6 Entropy measures

A random variable's entropy quantifies whether it has uncertainty or variance. The more uncertainty in the data, the higher the entropy value. Finding the entropy measurement's expression of the KM-UEHL will be the main aim in this sub-section. The formula for the Rényi entropy of *T* in mathematics is:

$$\bar{E}{}^{\circ}\left(\alpha \right)={\left(1-\alpha \right)}^{-1}\;\log\left(\int_{0}^{\infty }{\left(w\left(t\right)\right)}^{\alpha }dt\right),\alpha \neq 1,\alpha >0\text{.}$$

Based on Equation (6) and using exponential and binomial expansions, then ${\bar{E}}^{\circ }\left(\alpha \right)$ of the KM-UEHL distribution is

$$\bar{E}{}^{\circ}\left(\alpha \right)=\frac{1}{\left(1-\alpha \right)}\log\left\{\sum_{c_{1},c_{3}=0}^{\infty }\Delta_{c_{1},c_{2},c_{3}}\left(\alpha ,\varsigma \right)\kappa^{\alpha }\int_{0}^{1}t^{\alpha \left(\kappa -1\right)+c_{3}\kappa }{\left(1-t^{\kappa }\right)}^{\alpha \left(\varsigma -1\right)+\varsigma c_{2}}dt\right\},\Delta_{c_{1},c_{2},c_{3}}\left(\alpha ,\varsigma \right)=\sum_{c_{2}=0}^{c_{1}}\frac{{\left(-1\right)}^{c_{1}+c_{2}+c_{3}}\alpha^{c_{1}}}{c_{1}!}\left(\begin{array}{c} \alpha \left(\varsigma +1\right)+\varsigma c_{2}+c_{3}\\ c_{3} \end{array}\right)\left(\begin{array}{c} c_{1}\\ c_{2} \end{array}\right){\left(2e^{*}\varsigma \right)}^{\alpha }\text{.}$$

Let $z=t^{\kappa }\Rightarrow dz=\kappa t^{\kappa -1}dt$, then ${\bar{E}}^{\circ }\left(\alpha \right)$ has the expression:

$$\bar{E}{}^{\circ}\left(\alpha \right)={\left(1-\alpha \right)}^{-1}\;\log\left\{\sum_{c_{1},c_{3}=0}^{\infty }\kappa^{\alpha -1}\Delta_{c_{1},c_{2},c_{3}}\left(\alpha ,\varsigma \right)Β\left(\frac{\alpha \left(\kappa -1\right)+1}{\kappa }+c_{3},\alpha \left(\varsigma -1\right)+1+\varsigma c_{2}\right)\right\}\text{,}$$

The α −entropy of KM-UEHL distribution is given by:

$$I\left(\alpha \right)=\frac{1}{\alpha -1}\left[1-\int_{0}^{1}{\left(f\left(t\right)\right)}^{\alpha }dt\right],\alpha \neq 1,\alpha >0=\frac{1}{\alpha -1}\left[1-\left(\sum_{c_{1},c_{3}=0}^{\infty }\Delta_{c_{1},c_{2},c_{3}}\left(\alpha ,\varsigma \right)\kappa^{\alpha -1}Β\left(\frac{\alpha \left(\kappa -1\right)+1}{\kappa }+c_{3},\varsigma c_{2}+\alpha \left(\varsigma -1\right)+1\right)\right)\right]\text{.}$$

## 4 Estimation of parameters

Let *T*_1:m:n_, …, *T*_m:m:n_ be a PTII-CS from KM-UEHL distribution PDF given in Equation (6) with the censoring scheme ($R_{1}\text{,}$. . . , $R_{m}$). To simplify the notation (*t*_1_, …, *t*_m_) in place of (*t*_1:m:n_, …, *t*_m:m:n_) will be used. In this section, the estimation of the unknown parameters ς and κ based on ML and Bayesian methods is provided.

### 4.1 Maximum likelihood method

Under the PTII-CS, *T* = (*T*_1_, …, *T*_m_), the likelihood function for ϖ=(ς,κ) is given by

(20) $$L\left(\varpi \right)=C\prod_{i=1}^{m}\;w\left(\;t_{i}\right)\;{\left[1-W\left(t_{i}\right)\right]}^{R_{i}}\text{,}$$

where *C* = *n*(*n*− $R_{1}$ −1) ⋯ (*n*− $R_{1}$ −$R_{2}$ −⋯ −$R_{m-1}$ −*m*+1) (see Ref. [51]). Using Equations (5), (6) in Equation (20) provide:

$$L\left(\varpi \right)\propto {\left(\kappa \varsigma \right)}^{m}\prod_{i=1}^{m}\;{\left(1-t_{i}^{\kappa }\right)}^{\varsigma -1}t^{\kappa -1}e^{-\left[1-H_{i}\left(t_{i},\varpi \right)\right]}{\left(1+t_{i}^{\kappa }\right)}^{-\left(\varsigma +1\right)}\;{\left[1-e^{*}\left[1-e^{-\left[1-H_{i}\left(t_{i},\varpi \right)\right]}\right]\right]}^{R_{i}}\text{.}$$

The log-likelihood, namely $l^{*}\left(\varpi \right)\text{,}$ is given by:

$$l^{*}\left(\varpi \right)\propto m\;\ln\left(\varsigma \kappa \right)+\left(\varsigma -1\right)\sum_{i=1}^{m}\ln\left[1-t_{i}^{\kappa }\right]+\left(\kappa -1\right)\sum_{i=1}^{m}\ln\;t_{i}-\left(\varsigma +1\right)\sum_{i=1}^{m}\ln\left[1+t_{i}^{\kappa }\right]-\sum_{i=1}^{m}\left(1-H_{i}\left(t_{i},\kappa ,\varsigma \right)\right)+\sum_{i=1}^{m}R_{i}\;\ln\left[1-e^{*}\left[1-e^{-\left(1-H_{i}\left(t_{i},\kappa ,\varsigma \right)\right)}\right]\right]\text{,}$$

where $H_{i}\left(t_{i},\kappa ,\varsigma \right)={\left(\frac{1-t_{i}^{\kappa }}{1+t_{i}^{\kappa }}\right)}^{\varsigma },e^{*}=\frac{e}{e-1}\text{.}$ The appropriate likelihood equations, as usual in order, must be solved to obtain the ML estimates (MLEs) for ς and κ..

(21) $$\frac{\partial l^{*}\left(\varpi \right)}{\partial \varsigma }=\frac{m}{\varsigma }+\sum_{i=1}^{m}\ln\left[1-t_{i}^{\kappa }\right]+\sum_{i=1}^{m}H_{i}\left(t_{i},\kappa ,\varsigma \right)\ln\left[D_{i}\left(t_{i},\kappa \right)\right]-\sum_{i=1}^{m}\ln\left[1+t_{i}^{\kappa }\right]+\sum_{i=1}^{m}\frac{R_{i}e^{-\left(1-H_{i}\left(t_{i},\kappa ,\varsigma \right)\right)}H_{i}\left(t_{i},\kappa ,\varsigma \right)\ln\left[D_{i}\left(t_{i},\kappa \right)\right]}{1-e^{*}\left[1-e^{-\left(1-{Η}_{i}\left(t_{i},\kappa ,\varsigma \right)\right)}\right]}$$

(22) $$\frac{\partial l^{*}\left(\varpi \right)}{\partial \kappa }=\frac{m}{\kappa }-\sum_{i=1}^{m}\frac{\left(\varsigma -1\right)\ln\;t_{i}}{\left[t_{i}^{-\kappa }-1\right]}+\sum_{i=1}^{m}\ln\;t_{i}-\sum_{i=1}^{m}\frac{\left(\varsigma +1\right)\ln\;t_{i}}{\left[1+t_{i}^{-\kappa }\right]}-\sum_{i=1}^{m}\frac{\varsigma 2t_{i}^{\kappa }\;\ln\;t_{i}}{{\left(1+t_{i}^{\kappa }\right)}^{2}}{\left[D_{i}\left(t_{i},\kappa \right)\right]}^{\varsigma -1}-\;\sum_{i=1}^{m}\frac{e^{*}R_{i}e^{-\left(1-{Η}_{i}\left(t_{i},\kappa ,\varsigma \right)\right)}\varsigma 2t_{i}^{\kappa }\;\ln\;t_{i}{Η}_{i}\left(t_{i},\kappa ,\varsigma \right)}{\left\{1-e^{*}\left[1-e^{-\left(1-{Η}_{i}\left(t_{i},\kappa ,\varsigma \right)\right)}\right]\right\}\left(1-t_{i}^{2\kappa }\right)}\text{,}$$

where $D_{i}\left(t_{i},\kappa \right)=\left(\frac{1-t_{i}^{\kappa }}{1+t_{i}^{\kappa }}\right)$.

The aforementioned Equations (21), (22) cannot be analytically resolved in closed form. As a result, it is suggested to calculate the desired MLEs using some numerical approaches. The 'maxLik' package in R packages, which offers a straightforward implementation of the Newton-Raphson maximization method, can be readily employed. The MLEs of ς and κ for $R_{1}=R_{2}=$. . . = $R_{m-1}=0$ and $R_{m}=$ *n* − *m* are produced via TII-CS. Also, the MLEs of ς and κ are derived for $R_{1}=R_{2}=$. . . = $R_{m-1}=0$ and $R_{m}=$ 0 via complete dataset.

Asymptotic confidence bounds: Based on the asymptotic characteristics of the MLEs of the parameters, the ACIs of the parameters utilizing PTII-CS are established. To get CIs for the unknown parameters, one option is to use the asymptotic normal approximation. The asymptotic Fisher information matrix is obtained using Equations (21), (22) (see Appendix 1). Therefore, the asymptotic Fisher's information matrix can be written as:

$$I^{-1}\left(\varpi \right){=}_{{\left[\begin{array}{cc} \frac{-\partial^{2}l^{*}\left(\varpi \right)}{\partial \tau^{2}} & \frac{-\partial^{2}l^{*}\left(\varpi \right)}{\partial \tau \partial \kappa }\\ \frac{-\partial^{2}l^{*}\left(\varpi \right)}{\partial \kappa \partial \tau } & \frac{-\partial^{2}l^{*}\left(\varpi \right)}{\partial \kappa^{2}} \end{array}\right]}_{\begin{array}{c} \tau =\hat{\tau }\\ \kappa =\hat{\kappa } \end{array}}}$$

By computationally inverting the aforementioned Fisher's information matrix, the asymptotic variance-covariance matrix of the MLEs of the parameters may be calculated. It is known that the asymptotic distribution of ϖ, see Ref. [52], is given by:

$$\left(\hat{\kappa }-\kappa \right),\left(\hat{\varsigma }-\varsigma \right)∼N\left(\underline{0},I^{-1}\left(\kappa ,\varsigma \right)\right)$$

where $I^{-1}\left(\varpi \right)$ is the variance covariance matrix of set of parameters of KM-UEHL distribution based on PTII-CS. Therefore, the two-sided approximate (1−ε)% CIs for MLE of $\varpi ={\left(\kappa ,\varsigma \right)}^{T}$ can be obtained as follows:

$$a\text{nd}\;\begin{array}{l} L_{\kappa }=\hat{\kappa }-z_{\varepsilon /2}\sqrt{\text{var}\left(\hat{\kappa }\right)},U_{\kappa }=\hat{\kappa }+z_{\varepsilon /2}\sqrt{\text{var}\left(\hat{\kappa }\right)}\text{,}\\ L_{\varsigma }=\hat{\varsigma }-z_{\varepsilon /2}\sqrt{\text{var}\left(\hat{\varsigma }\right)},U_{\varsigma }=\hat{\varsigma }+z_{\varepsilon /2}\sqrt{\text{var}\left(\hat{\varsigma }\right),} \end{array}$$

where $z_{\varepsilon /2}$ is the 100(1−ε)% th standard normal percentile and var(.) denote the diagonal elements of the variance covariance matrix corresponding to the model parameters.

### 4.2 Bayesian estimation method

This section uses distinct loss functions to construct the BEs for the parameters ς and κ of the KM-UEHL distribution, based on PTII-CS. The BEs of ς and κ under squared error loss function (SEL), are respectively, defined by:

Let the prior distribution of ς and κ, represented by, π(ς),π(κ) has an independent gamma distribution. One way to express the joint gamma prior density of ς and κ, is as follows:

(23) $$\pi \left(\varsigma ,\kappa \right)\propto \varsigma^{h_{1}-1}e^{-q_{1}\varsigma }\kappa^{h_{2}-1}e^{-q_{2}\kappa };h_{i},q_{i}>0,i=1,2\text{.}$$

Eliciting hyper-parameters: The determination of hyper-parameters relies on the use of informative priors. These informative priors are obtained by setting the mean and variance of ς and κ equal to the mean and variance of the specified priors (Gamma priors) for ς and κ from the MK-UEHL distribution. Consequently, following [53], by equating the mean and variance of $\hat{\varsigma }$ and $\hat{\kappa }$ to the mean and variance of gamma priors, give,

$$\frac{1}{l}\sum_{j=1}^{l}{\hat{\varpi }}_{i}^{j}=\frac{h_{i}}{q_{i}},\frac{1}{l-1}{\sum_{j=1}^{l}\left[{\hat{\varpi }}_{i}^{j}-\frac{1}{l}\sum_{j=1}^{l}{\hat{\varpi }}_{i}^{j}\right]}^{2}=\frac{h_{i}}{q_{i}^{2}},i=1,2\;\hat{\varpi }=\left(\hat{\varsigma },\hat{\kappa }\right)\text{,}$$

l is the number of samples iteration. Now on solving the above two equations, the estimated hyper-parameters can be written as:

$$h_{i}=\frac{{\left[l^{-1}\sum_{j=1}^{l}{\hat{\varpi }}_{i}^{j}\right]}^{2}}{\;{\left(l-1\right)}^{-1}{\sum_{j=1}^{l}\left[{\hat{\varpi }}_{i}^{j}-\frac{1}{l}\sum_{j=1}^{l}{\hat{\varpi }}_{i}^{j}\right]}^{2}},q_{i}=\frac{l^{-1}\sum_{j=1}^{l}{\hat{\varpi }}_{i}^{j}}{\;{\left(l-1\right)}^{-1}{\sum_{j=1}^{l}\left[{\hat{\varpi }}_{i}^{j}-\frac{1}{l}\sum_{j=1}^{l}{\hat{\varpi }}_{i}^{j}\right]}^{2}}\text{.}$$

It is possible to determine the joint posterior of the KM-UEHL with parameters ς and κ as follows:

$$\pi \left(\varsigma ,\kappa |\underline{t}\right)=C^{-1}L\left(\varpi \right)\pi \left(\kappa ,\varsigma \right),C=\int_{0}^{\infty }\int_{0}^{\infty }L\left(\varpi \right)\pi \left(\kappa ,\varsigma \right)d\varsigma d\kappa \text{,}$$

which can be written as follows by using Equations (20), (23):

(24) $$\pi \left(\varsigma ,\kappa |\underline{t}\right)\propto \varsigma^{h_{1}+m-1}\;\kappa^{h_{2}+m-1}\prod_{i=1}^{m}\frac{t^{\kappa -1}{\left(1-t_{i}^{\kappa }\right)}^{\varsigma -1}}{{\left(1+t_{i}^{\kappa }\right)}^{\varsigma +1}}\;\exp-\left(q_{1}\varsigma +q_{2}\kappa -{Η}_{i}\left(t_{i},\kappa ,\varsigma \right)\right)\times {\left[1-e^{*}\left[1-{Η}_{i}\left(t_{i},\kappa ,\varsigma \right)\right]\right]}^{R_{i}}\text{.}$$

Analytically dealing with the joint posterior distribution is not feasible, as seen. The more adaptable Metropolis within Gibbs samplers and Gibbs sampling are useful MCMC subclasses. Hence, in order to create MCMC samples and acquire the Bayes estimates of ς and κ, the Metropolis-Hastings (M − H) approach with Gibbs sampling is used. Before employing the MCMC approach, it's essential to derive the complete conditional distributions for ς and κ. With Equation (24) in mind, the required full conditional distributions can be calculated as outlined below:

$$\pi \left(\varsigma |\kappa ,\underline{t}\right)\propto \varsigma^{h_{1}+m-1}\;\prod_{i=1}^{m}\frac{{\left(1-t_{i}^{\kappa }\right)}^{\varsigma -1}}{{\left(1+t_{i}^{\kappa }\right)}^{\varsigma +1}}\;\exp-\left(q_{1}\varsigma -{Η}_{i}\left(t_{i},\kappa ,\varsigma \right)\right)\;{\left[1-e^{*}\left[1-{Η}_{i}\left(t_{i},\kappa ,\varsigma \right)\right]\right]}^{R_{i}}\text{,}$$

and

$$\pi \left(\kappa |\varsigma ,\underline{t}\right)\propto \kappa^{h_{2}+m-1}\prod_{i=1}^{m}\frac{t^{\kappa -1}{\left(1-t_{i}^{\kappa }\right)}^{\varsigma -1}}{{\left(1+t_{i}^{\kappa }\right)}^{\varsigma +1}}\;\exp-\left(q_{2}\kappa -{Η}_{i}\left(t_{i},\kappa ,\varsigma \right)\right)\;{\left[1-e^{*}\left[1-{Η}_{i}\left(t_{i},\kappa ,\varsigma \right)\right]\right]}^{R_{i}}\text{.}$$

The following MH-within-Gibbs sampling steps can be used to obtain samples of and.

Step 1 Set the initial values $\varsigma^{\left(0\right)}=\hat{\varsigma }$ and $\kappa^{\left(0\right)}=\hat{\kappa }$

Step 2 *Set*I = 1.

Step 3 Generate $\varsigma^{*}\text{,}$ and $\kappa^{*}$ from $N\left(\hat{\varsigma },V_{\hat{\varsigma }}\right)\text{,}$ and $N\left(\hat{\kappa },V_{\hat{\kappa }}\right)\text{,}$ respectively.

Step 4 Obtain $\hbar_{\varsigma }=\min\left[1,\frac{\pi \left(\varsigma^{*}|\kappa^{\left(I-1\right)},t\right)}{\pi \left(\varsigma^{\left(I-1\right)}|\kappa^{\left(I-1\right)},t\right)}\right]\text{,}$ and $\hbar_{\kappa }=\min\left[1,\frac{\pi \left(\kappa^{*}|\varsigma^{\left(I-1\right)},t\right)}{\pi \left(\kappa^{\left(I-1\right)}|\varsigma^{\left(I-1\right)},t\right)}\right]\text{.}$

Step 5 Generate samples $U_{j};j=1,2$ from the uniform *U*(0, 1) distribution.

Step 6 If $U_{1}\leq \hbar_{\varsigma }\text{,}$ and $U_{2}\leq \hbar_{\kappa }\text{,}$ then set $\varsigma^{\left(I\right)}=\varsigma^{*},\kappa^{\left(I\right)}=\kappa^{*}$; otherwise $\varsigma^{\left(I\right)}=\varsigma^{\left(I-1\right)}\text{,}$ and $\kappa^{\left(I\right)}=\kappa^{\left(I-1\right)}\text{,}$ respectively.

Step 7 *Set*I = *I*+ 1.

Step 8 Repeat steps 3–7 *B* times and obtain $\varsigma^{\left(I\right)}\text{,}$ and $\kappa^{\left(I\right)}\text{,}$ for *I* = 1, 2, …, *B.*

The BEs are obtained via SEL. The 95 % two-sided HPD credible interval for the unknown parameters or any function of them is given $\left[\Psi_{0.025N:N},\Psi_{0.975N:N}\right]$ by using the method proposed by Ref. [54].

## 5 Predication

### 5.1 Non-Bayesian two-sample prediction

Let $X_{1:m:n}^{\left(R_{1},R_{2},{\ldots ,R}_{m}\right)},X_{2:m:n}^{\left(R_{1},R_{2},{\ldots ,R}_{m}\right)},\ldots ,X_{m:m:n}^{\left(R_{1},R_{2},{\ldots ,R}_{m}\right)}\;b$ e a Type II ordered statistics that have been progressively censored from a sample of size n using the censoring strategy $R_{1},R_{2},...,R_{m}$ from a continuous distribution. Assume that $Z_{1:m:n}^{\left(S_{1},S_{2},{\ldots ,S}_{m}\right)},Z_{2:m:n}^{\left(S_{1},S_{2},\ldots ,{\;S}_{m}\right)},\ldots ,Z_{m:m:n}^{\left(S_{1},S_{2},{\ldots ,S}_{m}\right)}$ is another (unobserved) independent PTII-CS ordered statistics of size *m* from a sample of size *n* with progressive censoring scheme, $\left(S_{1},S_{2},...,S_{m}\right)\text{,}$ according to Ref. [55]. While the second sample is thought of as the "future", the first sample is thought of as "informative" (history). Assume that in the future sample of size *m*, 1≤s≤n, $Z_{S}$ represents the sth order statistic. The issue of prediction is crucial in practice, especially when choosing the best experiments to do (see [54], and [56] for more information). Our goal in this article is to predict $Z_{S}$ from the future sample. If s=1,2,...,m, the PDF of $Z_{S}$ is derived as

(25) $$f\left(z_{s}|\varpi \right)=\frac{m!}{\left(m-1\right)!\left(n-m\right)!}f\left(z_{s}\right){\left[F\left(z_{s}\right)\right]}^{m-1}{\left[1-F\left(z_{s}\right)\right]}^{n-m}=\frac{m!}{\left(m-1\right)!\left(n-m\right)!}\frac{2e^{*}\kappa \varsigma z_{s}^{\kappa -1}{\left(1-z_{s}^{\kappa }\right)}^{\varsigma -1}}{{\left(1+z_{s}^{\kappa }\right)}^{\varsigma +1}}e^{-\left[1-H_{S}\left(z_{S},\varpi \right)\right]}\;{\left\{e^{*}\left[1-e^{-\left[1-H_{s}\left(z_{s},\varpi \right)\right]}\right]\right\}}^{m-1}\times {\left\{1-e^{*}\left[1-e^{-\left[1-H_{s}\left(z_{s},\varpi \right)\right]}\right]\right\}}^{n-m},1\;<s<n\text{,}$$

where, $H_{s}\left(z_{s},\varpi \right)={\left(\frac{1-z_{s}^{\kappa }}{1+z_{s}^{\kappa }}\right)}^{\varsigma }\text{.}$ The MLE of $Z_{S}$, given $\underline{t}$, may be calculated using the conditional PDF of the sth order statistic, which is provided by Equation (25) after replacing the parameters (ς,κ) by their MLE $\left(\hat{\varsigma },\hat{\kappa }\right)$, assuming that the parameters (ς,κ) of the KM-UEHL distribution are unknown and independent.

$${\hat{z}}_{\left(s\right)ML}=E(Z_{s}|\varpi )=\frac{m!2e^{*}\kappa \varsigma }{\left(m-1\right)!\left(n-m\right)!}\int_{0}^{1}z_{s}^{\kappa }\frac{{\left(1-z_{s}^{\kappa }\right)}^{\varsigma -1}}{{\left(1+z_{s}^{\kappa }\right)}^{\varsigma +1}}e^{-\left[1-H_{s}\left(z_{s},\varpi \right)\right]}\;{\left\{e^{*}\left[1-e^{-\left[1-H_{s}\left(z_{s},\varpi \right)\right]}\right]\right\}}^{m-1}\times {\left\{1-e^{*}\left[1-e^{-\left[1-H_{s}\left(z_{s},\varpi \right)\right]}\right]\right\}}^{n-m}\;dz$$

The two-sided 100(1−ε)% ML prediction interval (MLPI) for future observation $Z_{S}>0$ is provided by:

$$P\left({L\leq Z}_{S}\leq U\right)=1-\varepsilon \text{,}$$

where lower bound (L) and upper bound (U) may be derived by numerically resolving the two following equations:

$$P\left(Z_{S}\geq L|\underline{t}\right)=1-\frac{\varepsilon }{2},P\left(Z_{S}\geq U|\underline{t}\right)=\frac{\varepsilon }{2}$$

### 5.2 Bayesian two-samples prediction

This sub-section introduces the posterior predictive distribution of the unobserved lifetimes at the failure time and suggests an MCMC method to generate samples from its posterior distribution. This enables to compute the appropriate BEs. The joint posterior distribution of $Z_{S},\varsigma$ and κ is thus given by:

$$\begin{array}{c} \pi \left(z_{s},\varsigma ,\kappa |\underline{t}\right)=f\left(z_{s}|,\varsigma ,\kappa \right)\pi \left(\varsigma ,\kappa |\underline{t}\right)=\frac{m!}{\left(m-1\right)!\left(n-m\right)!}\frac{2e^{*}\kappa^{h_{2}}\varsigma^{h_{1}}e^{1-q_{1}\varsigma }z_{s}^{\kappa -1}e^{-q_{2}\kappa }{\left(1-z_{s}^{\kappa }\right)}^{\varsigma -1}}{{\left(1+z_{s}^{\kappa }\right)}^{\varsigma +1}}e^{-\left[1-H_{s}\left(z_{s},\varpi \right)\right]}\\ {\left\{e^{*}\left[1-e^{-\left[1-H_{s}\left(z_{s},\varpi \right)\right]}\right]\right\}}^{m-1}\;{\left\{1-e^{*}\left[1-e^{-\left[1-H_{s}\left(z_{s},\varpi \right)\right]}\right]\right\}}^{n-m}\;\text{.} \end{array}$$

The unobserved lifetime's posterior predictive distribution, *Z*_*S*_, is represented by the expression $\pi \left(z_{s}|\underline{t}\right)=\int_{0}^{\infty }\int_{0}^{\infty }\pi \left(z_{s},\varsigma ,\kappa |\underline{t}\right)d\varsigma d\kappa \text{.}$ It is impossible to determine the posterior predictive distribution, $\pi \left(z_{s}|\underline{t}\right)\text{.}$ As a result, the BE under the SEL of the system cannot be determined analytically. Then, the MCMC technique is used to sample the posterior density function and subsequently calculate the BEs. The Bayesian predictive density function of *Z*_*S*_ is:

$$H\left(z_{s}|\varsigma ,\kappa \right)=\int_{0}^{\infty }\int_{0}^{\infty }f\left(z_{s}|\varsigma ,\kappa \right)\pi \left(z_{s},\varsigma ,\kappa |\underline{t}\right)d\varsigma d\kappa \text{.}$$

The two-sided 100(1−ε)% Bayesian prediction interval (BPI) for $Z_{S}>0$ is provided by:

$$P\left({L\leq Z}_{S}\leq U\right)=1-\varepsilon \text{,}$$

where L and U may be derived by numerically resolving the two following equations:

$$P\left(Z_{S}\geq L|\underline{t}\right)=1-\frac{\varepsilon }{2},P\left(Z_{S}\geq U|\underline{t}\right)=\frac{\varepsilon }{2}\text{.}$$

In this part, the MCMC approach is used to induce Bayes two-sample predictions; see Ref. [57]. The forms can be used to approximate the predictive PDF using the MCMC approach

$$h\left(z_{s}|\underline{t}\right)\approx \frac{\sum_{p=1}^{N}f\left(z_{s}|\varsigma_{p},\kappa_{p};\underline{t}\right)}{N\sum_{p=1}^{N}\int_{0}^{\infty }f\left(z_{s}|\varsigma_{p},\kappa_{p};\underline{t}\right)dz_{s}}\text{.}$$

where $\varsigma_{p}$ and $\kappa_{p}$ are produced from the posterior density function, along with *p* = 1, 2,.., N. The following two nonlinear equations may be solved numerically to determine the two sides 100(1−ε)% BPI (L,U) of the future observation $Z_{S}$.

$$\frac{\sum_{p=1}^{N}\int_{L}^{\infty }f\left(z_{s}|\varsigma_{p},\kappa_{p};\underline{t}\right)dz_{s}}{N\sum_{p=1}^{N}\int_{0}^{\infty }f\left(z_{s}|\varsigma_{p},\kappa_{p};\underline{t}\right)dz_{s}}=1-\frac{\varepsilon }{2},\frac{\sum_{p=1}^{N}\int_{U}^{\infty }f\left(z_{s}|\varsigma_{p},\kappa_{p};\underline{t}\right)dz_{s}}{N\sum_{p=1}^{N}\int_{0}^{\infty }f\left(z_{s}|\varsigma_{p},\kappa_{p};\underline{t}\right)dz_{s}}=\frac{\varepsilon }{2}\text{.}$$

In order to solve the aforementioned equations and determine L and U for a given ε, numerical techniques are often required.

## 6 Numerical Illustration

In this section, some experimental findings are offered that show how the proposed model behaves when various estimate techniques are applied to various sample sizes, sample sizes that have been censored, and various sampling schemes.

The simulation studies were conducted with the following objectives.

• To assess the effectiveness of MK-UEHL using PTII-CS in the context of two-sample prediction.
• Simulation studies yield empirical results for particular scenarios. Therefore, these studies frequently encompass multiple data-generation methods to encompass a variety of scenarios.
• To evaluate and compare the performance of the suggested estimation techniques by examining their simulated mean squared errors (MSEs), biases, and average CIs.

Various sample sizes are taken into account as *n* = 30, 50, and 100. Different censored sample sizes as *m* = 20, 26 at *n* = 30, *m* = 34, 46 at *n* = 50 and *m* = 74, 90 at *n* = 100 are selected. Three sampling schemes (Sch.) are specified as.

Sch. 1: $R_{i}=2;i=1,\ldots ,\frac{n-m}{2},R_{j}=0;j=\frac{n-m}{2}+1,\ldots ,m$.

Sch. 2: $R_{i}=0;i=2,\ldots ,m,R_{1}=n-m$.

Sch. 3: $R_{i}=0;i=1,\ldots ,m-1,R_{m}=n-m$.

In Sch. 3, at the initial failure time point, the *n* and *m* remaining units are eliminated. It should be noted that for fixed *n* and *m*, the estimated experiment time is maximum for the TII-CS, which is the reverse of Type II, and least for the TII-CS. When the parameters are taken to have actual values of (ς,κ) = (0.6, 0.4), (0.6, 2), and (2, 2), 10,000 times PTII-CS is replicated from a KM-UEHL distribution. The behavior of various estimating methodologies is difficult to compare conceptually, so comprehensive simulation studies are conducted to assess the behavior of various estimates using bias, MSE, and length of CI (LCI) criteria (for ACI, the LCI can be denoted as LACI; for HPD credible interval, the LCI can be denoted as LCCI). Also, the prediction point is estimated for different values of *k* and obtained the lower and upper values.

This section also focuses on calculating the MLE and BPIs for future lifetimes, as well as their actual (simulated) prediction levels, using a PTII-CS model. The scenario that reflects the usual order statistics, will take into consideration, even the predictive interval of the sth future lifetime in a future increasingly TII-CS is constructed. Only predictions are made for the two future ordered lifetimes that are practically of particular importance. The following methods are followed to determine the 95 % MLE and Bayesian prediction boundaries for the future order statistics $Z_{S}$, as well as their actual (simulated) prediction levels.

For a future progressively ordered statistic from the same population, Bayesian prediction bounds are shown using PTII-censored informative data from the KM-UEHL distribution. This sample technique is a common one, as are the article's findings. The TII-CS, for which Sch. 3 of the PTII- CS.

The outcomes of the recommended methods for estimating point and interval parameters are presented in Table 2, Table 3, Table 4. These results offer valuable insights and are discussed in the following comments.

⁃ As the sample size (*n*) increases, the MSE, bias, and LCI for both estimates of parameters ς and κ tend to diminish. This observation underscores the consistency property of these estimates as the sample size requirements are augmented.
⁃ The parameter estimates are derived from the most optimal unbiased estimator when the MSE and bias values approach zero.
⁃ As the size of the censored sample (*m*) increases, the estimators' measures (bias, MSE, and LCI) tend to decrease significantly, approaching values close to zero for all methods.
⁃ Among Schemes 1, 2, and 3, Sch. 2 exhibits the most modest values for the estimators' measures.
⁃ With a constant parameter ς and the actual value of κ rises, the MSE, bias, and LCI for both parameter estimates decrease.
⁃ The MSE and length of CI for BEs are smaller than the MLE for all true parameter values.
⁃ As the size of prediction *k* grows, the difference between upper and lower for each estimate nears zero.
⁃ Bayesian prediction is better than MLE predication.
⁃ Simulations are a valuable tool for understanding and predicting the epidemiological behavior of COVID-19. They offer insights into the effectiveness of interventions, identify high-risk groups, and explore different scenarios to prepare for potential future developments.
⁃ By simulating the potential impact of new variants with different transmissibility or disease severity, public health officials can develop contingency plans, resource allocation strategies beforehand, and to obtain mathematical distribution for these variants.
⁃ Simulations can predict potential increases in hospitalizations based on trends in new cases. This allows healthcare systems to surge staffing and resources in anticipation, improving their capacity to handle a potential influx of patients.

**Table 2:** Parameters and predictors by ML and Bayesian where ς=0.6,κ=0.4.

				Parameters							Prediction					
				MLE			Bayesian				MLE			Bayesian		
Sch.	n	m		Bias	MSE	LACI	Bias	MSE	LCCI	k	$z_{S}$	L	U	$z_{S}$	L	U
1	30	20	ς	0.1376	0.0552	0.8630	0.0083	0.0100	0.3921	22	0.6963	0.4623	0.8875	0.7235	0.5661	0.8871
			κ	0.1392	0.0255	0.5865	0.0342	0.0066	0.3029		0.9365	0.8173	0.9993	0.9563	0.9000	0.9973
		26	ς	0.1001	0.0345	0.6895	0.0034	0.0046	0.2614	28	0.7038	0.5128	0.8867	0.7213	0.5928	0.8398
			κ	0.1186	0.0229	0.5629	0.0126	0.0031	0.2141		0.9433	0.8547	0.9958	0.9582	0.9176	0.9891
	50	34	ς	0.0780	0.0252	0.5945	0.0031	0.0037	0.2320	40	0.8057	0.6606	0.9461	0.8222	0.7051	0.9177
			κ	0.0771	0.0122	0.4167	0.0123	0.0030	0.2029		0.9732	0.9259	0.9990	0.9804	0.9507	0.9969
		46	ς	0.0531	0.0162	0.4833	0.0058	0.0032	0.2129	48	0.8123	0.6767	0.9295	0.8229	0.7438	0.9007
			κ	0.0657	0.0111	0.4009	0.0146	0.0023	0.1783		0.9764	0.9358	0.9982	0.9818	0.9646	0.9946
	100	74	ς	0.0335	0.0082	0.3470	0.0042	0.0030	0.2021	80	0.8232	0.7308	0.9251	0.8274	0.7501	0.8933
			κ	0.0413	0.0047	0.2615	0.0271	0.0019	0.1571		0.9759	0.9489	0.9971	0.9781	0.9579	0.9923
		90	ς	0.0256	0.0068	0.3174	0.0075	0.0017	0.1569	95	0.8258	0.7337	0.9062	0.8296	0.7784	0.8856
			κ	0.0379	0.0044	0.2536	0.0143	0.0011	0.1257		0.9769	0.9519	0.9949	0.9792	0.9667	0.9908
2	30	20	ς	0.1092	0.0412	0.7533	0.0080	0.0097	0.3708	22	0.6973	0.4718	0.9003	0.7213	0.5715	0.8756
			κ	0.0959	0.0195	0.5262	0.0253	0.0068	0.2990		0.9394	0.8348	0.9983	0.9561	0.8992	0.9923
		26	ς	0.0878	0.0310	0.6591	0.0023	0.0042	0.2548	28	0.7064	0.5063	0.8871	0.7238	0.6070	0.8404
			κ	0.1110	0.0212	0.5436	0.0123	0.0033	0.2212		0.9453	0.8624	0.9984	0.9593	0.9267	0.9897
	50	34	ς	0.0613	0.0196	0.5303	0.0021	0.0041	0.2306	40	0.8075	0.6557	0.9421	0.8207	0.7113	0.9207
			κ	0.0580	0.0116	0.4123	0.0112	0.0030	0.2127		0.9746	0.9325	0.9999	0.9803	0.9542	0.9970
		46	ς	0.0502	0.0158	0.4783	0.0088	0.0029	0.2111	48	0.8132	0.6757	0.9212	0.8212	0.7463	0.8923
			κ	0.0621	0.0103	0.3869	0.0116	0.0022	0.1755		0.9769	0.9412	0.9986	0.9816	0.9662	0.9939
	100	74	ς	0.0390	0.0077	0.3309	0.0078	0.0028	0.2040	80	0.8200	0.7200	0.9013	0.8265	0.7524	0.8894
			κ	0.0376	0.0052	0.2762	0.0247	0.0020	0.1650		0.9754	0.9479	0.9958	0.9780	0.9585	0.9915
		90	ς	0.0268	0.0067	0.3156	0.0087	0.0017	0.1572	95	0.8242	0.7392	0.9039	0.8290	0.7693	0.8771
			κ	0.0305	0.0044	0.2551	0.0138	0.0011	0.1275		0.9767	0.9534	0.9963	0.9791	0.9652	0.9905
3	30	20	ς	0.1776	0.0869	1.0782	0.0057	0.0108	0.3873	22	0.6930	0.4424	0.9000	0.7257	0.5665	0.8835
			κ	0.1581	0.0267	0.5906	0.0314	0.0063	0.2902		0.9319	0.8016	0.9996	0.9568	0.8998	0.9958
		26	ς	0.1257	0.0493	0.8190	0.0044	0.0044	0.2552	28	0.6979	0.4806	0.9080	0.7193	0.5987	0.8292
			κ	0.1244	0.0238	0.5728	0.0065	0.0034	0.2309		0.9387	0.8317	0.9995	0.9580	0.9211	0.9878
	50	34	ς	0.0947	0.0313	0.6571	0.0042	0.0038	0.2333	40	0.8021	0.6423	0.9359	0.8206	0.7057	0.9283
			κ	0.0837	0.0119	0.4077	0.0063	0.0031	0.2297		0.9715	0.9141	0.9997	0.9799	0.9510	0.9972
		46	ς	0.0552	0.0152	0.4661	0.0113	0.0033	0.2228	48	0.8124	0.6849	0.9246	0.8200	0.7407	0.9014
			κ	0.0732	0.0100	0.3743	0.0116	0.0022	0.1777		0.9765	0.9404	0.9982	0.9812	0.9633	0.9951
	100	74	ς	0.0340	0.0109	0.4011	0.0103	0.0030	0.2184	80	0.8233	0.7200	0.9219	0.8282	0.7480	0.8899
			κ	0.0357	0.0055	0.2844	0.0226	0.0019	0.1610		0.9755	0.9454	0.9977	0.9783	0.9590	0.9922
		90	ς	0.0227	0.0072	0.3287	0.0084	0.0019	0.1641	95	0.8262	0.7412	0.9169	0.8293	0.7804	0.8882
			κ	0.0297	0.0048	0.2686	0.0143	0.0011	0.1233		0.9771	0.9495	0.9950	0.9791	0.9651	0.9898

**Table 3:** Parameters and predictors by ML and Bayesian where ς=0.6,κ=2.

				MLE			Bayesian				MLE			Bayesian		
Sch.	n	m		Bias	MSE	LACI	Bias	MSE	LCCI	k	$z_{S}$	L	U	$z_{S}$	L	U
1	30	20	ς	0.1409	0.0512	0.8236	0.0086	0.0096	0.3706	22	0.9274	0.8678	0.9801	0.9351	0.8895	0.9750
			κ	0.1379	0.5582	2.7232	0.0030	0.0205	0.5381		0.9866	0.9635	0.9996	0.9908	0.9784	0.9992
		26	ς	0.0870	0.0333	0.6857	0.0057	0.0065	0.2673	28	0.9307	0.8787	0.9790	0.9358	0.9051	0.9671
			κ	0.0944	0.4987	2.6689	0.0012	0.0198	0.3447		0.9884	0.9709	0.9994	0.9913	0.9833	0.9981
	50	34	ς	0.0791	0.0245	0.5851	0.0047	0.0062	0.3124	40	0.9567	0.9213	0.9888	0.9600	0.9316	0.9827
			κ	0.0716	0.2583	1.9127	0.0009	0.0163	0.5460		0.9945	0.9850	0.9999	0.9959	0.9905	0.9996
		46	ς	0.0372	0.0140	0.4551	0.0046	0.0036	0.2312	48	0.9598	0.9309	0.9855	0.9612	0.9396	0.9783
			κ	0.0517	0.2545	1.9367	0.0004	0.0158	0.3422		0.9955	0.9892	0.9997	0.9962	0.9927	0.9992
	100	74	ς	0.0343	0.0087	0.3571	0.0045	0.0032	0.2114	80	0.9612	0.9391	0.9843	0.9620	0.9442	0.9782
			κ	0.0328	0.1226	1.3489	0.0004	0.0148	0.5282		0.9950	0.9892	0.9995	0.9955	0.9915	0.9985
		90	ς	0.0236	0.0066	0.3135	0.0041	0.0023	0.1837	95	0.9620	0.9412	0.9829	0.9630	0.9458	0.9760
			κ	0.0269	0.1136	1.3048	0.0002	0.0074	0.3340		0.9953	0.9903	0.9993	0.9958	0.9922	0.9983
2	30	20	ς	0.1244	0.0497	0.8238	0.0076	0.0097	0.3750	22	0.9283	0.8637	0.9828	0.9353	0.8917	0.9761
			κ	0.1201	0.6269	2.9591	0.0045	0.0214	0.5583		0.9870	0.9624	0.9996	0.9908	0.9793	0.9992
		26	ς	0.1088	0.0352	0.6897	0.0143	0.0047	0.2703	28	0.9304	0.8735	0.9771	0.9360	0.9027	0.9643
			κ	0.1385	0.5609	2.7288	0.0009	0.0085	0.3466		0.9881	0.9685	0.9997	0.9914	0.9835	0.9981
	50	34	ς	0.0770	0.0259	0.6047	0.0111	0.0036	0.2989	40	0.9573	0.9184	0.9906	0.9604	0.9322	0.9824
			κ	0.0868	0.3340	2.1621	0.0003	0.0072	0.5591		0.9946	0.9836	0.9998	0.9960	0.9901	0.9993
		46	ς	0.0513	0.0155	0.4724	0.0050	0.0034	0.2328	48	0.9592	0.9298	0.9857	0.9612	0.9405	0.9786
			κ	0.0715	0.2741	1.9752	0.0003	0.0070	0.3367		0.9953	0.9880	0.9996	0.9962	0.9927	0.9993
	100	74	ς	0.0282	0.0078	0.3397	0.0093	0.0030	0.2108	80	0.9618	0.9356	0.9821	0.9624	0.9436	0.9780
			κ	0.0394	0.1210	1.3289	0.0005	0.0068	0.5197		0.9952	0.9892	0.9993	0.9956	0.9915	0.9986
		90	ς	0.0262	0.0073	0.3292	0.0025	0.0024	0.1856	95	0.9624	0.9381	0.9804	0.9631	0.9463	0.9776
			κ	0.0432	0.1205	1.3186	0.0023	0.0061	0.3217		0.9953	0.9899	0.9993	0.9958	0.9924	0.9986
3	30	20	ς	0.1728	0.0801	1.0330	0.0144	0.0107	0.3988	22	0.9270	0.8564	0.9805	0.9340	0.8908	0.9795
			κ	0.1524	0.6568	2.9451	0.0042	0.0212	0.5587		0.9855	0.9550	0.9998	0.9905	0.9783	0.9992
		26	ς	0.1253	0.0437	0.7650	0.0131	0.0050	0.2802	28	0.9289	0.8650	0.9769	0.9361	0.9017	0.9662
			κ	0.1398	0.6611	2.9946	0.0008	0.0087	0.3613		0.9874	0.9650	0.9998	0.9914	0.9834	0.9986
	50	34	ς	0.0870	0.0336	0.6891	0.0162	0.0047	0.3247	40	0.9569	0.9189	0.9898	0.9599	0.9316	0.9853
			κ	0.0843	0.3353	2.1725	0.0005	0.0062	0.5567		0.9943	0.9834	0.9998	0.9958	0.9897	0.9995
		46	ς	0.0405	0.0145	0.4621	0.0058	0.0036	0.2355	48	0.9599	0.9289	0.9868	0.9612	0.9376	0.9775
			κ	0.0623	0.2472	1.8880	0.0001	0.0057	0.3361		0.9955	0.9884	0.9997	0.9962	0.9924	0.9993
	100	74	ς	0.0388	0.0112	0.4056	0.0143	0.0034	0.2186	80	0.9609	0.9327	0.9821	0.9620	0.9422	0.9777
			κ	0.0333	0.1259	1.3668	0.0010	0.0042	0.5232		0.9949	0.9878	0.9993	0.9955	0.9912	0.9987
		90	ς	0.0389	0.0080	0.3387	0.0071	0.0026	0.1933	95	0.9614	0.9390	0.9803	0.9628	0.9472	0.9785
			κ	0.0330	0.1208	1.3492	0.0027	0.0037	0.3422		0.9951	0.9896	0.9989	0.9957	0.9923	0.9985

**Table 4:** Parameters and predictors by ML and Bayesian where ς=2,κ=2.

				MLE			Bayesian				MLE			Bayesian		
Sch.	n	m		Bias	MSE	LACI	Bias	MSE	LCCI	k	$z_{S}$	L	U	$z_{S}$	L	U
1	30	20	ς	0.2276	1.3746	4.2375	0.0020	0.0292	0.5793	22	0.6720	0.5618	0.7773	0.6831	0.6347	0.7273
			κ	0.0840	0.2897	2.0053	0.0040	0.0185	0.5518		0.8258	0.7122	0.9197	0.8430	0.8094	0.8788
		26	ς	0.1618	0.7820	3.2275	0.0019	0.0278	0.3364	28	0.6768	0.5904	0.7703	0.6843	0.6557	0.7162
			κ	0.0759	0.2456	1.8505	0.0020	0.0176	0.3339		0.8313	0.7386	0.9075	0.8435	0.8239	0.8668
	50	34	ς	0.0980	0.4683	2.5714	0.0018	0.0200	0.3154	40	0.7316	0.6533	0.8178	0.7370	0.6939	0.7789
			κ	0.0431	0.1440	1.4493	0.0018	0.0171	0.3024		0.8736	0.7976	0.9390	0.8816	0.8519	0.9097
		46	ς	0.0876	0.3439	2.1948	0.0006	0.0077	0.3334	48	0.7316	0.6581	0.8014	0.7366	0.7086	0.7640
			κ	0.0445	0.1306	1.3737	0.0017	0.0072	0.3315		0.8741	0.8117	0.9336	0.8814	0.8626	0.8997
	100	74	ς	0.0459	0.1491	1.4708	0.0005	0.0071	0.2514	80	0.7375	0.6790	0.7868	0.7416	0.6983	0.7748
			κ	0.0247	0.0649	0.9803	0.0016	0.0069	0.4609		0.8712	0.8260	0.9173	0.8761	0.8494	0.9037
		90	ς	0.0456	0.1263	1.3474	0.0004	0.0070	0.2086	95	0.7377	0.6855	0.7877	0.7406	0.7172	0.7673
			κ	0.0272	0.0556	0.8997	0.0015	0.0060	0.2917		0.8712	0.8229	0.9111	0.8754	0.8574	0.8920
2	30	20	ς	0.2081	1.3255	4.2100	0.0050	0.0219	0.5808	22	0.6767	0.5560	0.7724	0.6849	0.6374	0.7329
			κ	0.0892	0.3155	2.0889	0.0015	0.0194	0.5354		0.8298	0.7204	0.9227	0.8442	0.8088	0.8784
		26	ς	0.1596	0.8202	3.3239	0.0010	0.0078	0.3381	28	0.6788	0.5908	0.7760	0.6840	0.6563	0.7168
			κ	0.0793	0.2629	1.9123	0.0010	0.0078	0.3422		0.8329	0.7382	0.9089	0.8434	0.8227	0.8650
	50	34	ς	0.1298	0.5475	2.7173	0.0006	0.0076	0.5154	40	0.7282	0.6536	0.8149	0.7359	0.6927	0.7788
			κ	0.0586	0.1652	1.5265	0.0007	0.0062	0.3256		0.8702	0.7931	0.9362	0.8810	0.8510	0.9078
		46	ς	0.0624	0.2459	1.8823	0.0005	0.0075	0.3391	48	0.7332	0.6669	0.8018	0.7353	0.7064	0.7625
			κ	0.0322	0.1116	1.2855	0.0002	0.0060	0.3206		0.8763	0.8192	0.9319	0.8805	0.8619	0.9001
	100	74	ς	0.0443	0.1464	1.4601	0.0004	0.0071	0.5087	80	0.7372	0.6814	0.7913	0.7404	0.6983	0.7769
			κ	0.0224	0.0653	0.9865	0.0002	0.0051	0.3145		0.8711	0.8262	0.9188	0.8754	0.8502	0.9063
		90	ς	0.0406	0.1159	1.2964	0.0003	0.0071	0.3158	95	0.7373	0.6886	0.7812	0.7395	0.7147	0.7662
			κ	0.0218	0.0603	0.9479	0.0002	0.0050	0.3029		0.8714	0.8284	0.9126	0.8746	0.8572	0.8927
3	30	20	ς	0.3373	2.6091	5.7559	0.0008	0.0723	0.6066	22	0.6725	0.5585	0.7869	0.6835	0.6321	0.7313
			κ	0.1197	0.4023	2.3036	0.0012	0.0186	0.5239		0.8226	0.7047	0.9400	0.8431	0.8056	0.8787
		26	ς	0.1827	0.9705	3.5881	0.0003	0.0683	0.3555	28	0.6788	0.5818	0.7700	0.6843	0.6538	0.7153
			κ	0.0882	0.2609	1.8803	0.0012	0.0091	0.3414		0.8317	0.7423	0.9204	0.8436	0.8208	0.8657
	50	34	ς	0.1685	0.9572	3.6022	0.0003	0.0230	0.5882	40	0.7278	0.6417	0.8116	0.7359	0.6950	0.7835
			κ	0.0619	0.1995	1.6830	0.0003	0.0073	0.2510		0.8686	0.7811	0.9435	0.8809	0.8505	0.9095
		46	ς	0.1019	0.3972	2.3391	0.0002	0.0074	0.3400	48	0.7311	0.6625	0.7960	0.7367	0.7122	0.7629
			κ	0.0512	0.1450	1.4385	0.0002	0.0069	0.2318		0.8733	0.8053	0.9323	0.8815	0.8632	0.8976
	100	74	ς	0.0726	0.2587	1.9117	0.0001	0.0062	0.2522	80	0.7347	0.6769	0.7934	0.7401	0.7061	0.7808
			κ	0.0278	0.0776	1.0708	0.0001	0.0051	0.2045		0.8684	0.8144	0.9245	0.8752	0.8497	0.9025
		90	ς	0.0426	0.1501	1.4820	0.0001	0.0046	0.2309	95	0.7371	0.6859	0.7904	0.7398	0.7150	0.7632
			κ	0.0181	0.0620	0.9660	0.0001	0.0041	0.1931		0.8712	0.8228	0.9165	0.8748	0.8580	0.8917

## 7 Data application

This section examines two genuine COVID-19 mortality rate datasets from Saudi Arabia and the United Kingdom to demonstrate how the MK-UEHL distribution can be applied practically. We selected datasets that.

• Contained daily or weekly COVID-19 mortality rates within the 0–1 range.
• Were obtained from reputable sources, such as government agencies or public health organizations.
• Were publicly available for reproducibility purposes.

Data preprocessing: The data were reviewed and examined to ensure that there are no missing values and that there are no outlier values, as the model used works on data with a range from 0 to 1. The KM-UEHL distribution's performance is compared with the following established models for modeling proportions or probabilities: UEHL, unit-Gompertz (UG), unit-Lindley (UL) [58], Topp-Leone (TL), unit generalized log Burr XII (UGLBXII) [59], unit exponential Pareto (UEP) [60], Kumaraswamy (Kw), Beta, unit Weibull (UW), and unit Burr-XII (UBXII). The specified models' unknown parameters were estimated using both estimation techniques. All the models are compared using the standard error (SE), Kolmogorov-Smirnov (K**) with P-value (PV-K**), the Cramer-von Mises (W**), and the Anderson-Darling (A**) statistics. The Akaike information value criterion (AIVC), Hannan-Quinn IVC (HQIVC), Bayesian IVC (BIVC), and consistent AIVC (CAIVC) are some examples of classic value criteria that are used to compare fitted models.

Data set I: The first set of data shows the United Kingdom's COVID-19 death rates for the 82 days between May 1, 2021, and July 16, 2021. The details are as follows.

0.0023, 0.0023, 0.0023, 0.0046, 0.0065, 0.0067, 0.0069, 0.0069, 0.0091, 0.0093, 0.0093, 0.0093, 0.0111, 0.0115, 0.0116, 0.0116, 0.0119, 0.0133, 0.0136, 0.0138, 0.0138, 0.0159, 0.0161, 0.0162, 0.0162, 0.0162, 0.0163, 0.0180, 0.0187, 0.0202, 0.0207, 0.0208, 0.0225, 0.0230, 0.0230, 0.0239, 0.0245, 0.0251, 0.0255, 0.0255, 0.0271, 0.0275, 0.0295, 0.0297, 0.0300, 0.0302, 0.0312, 0.0314, 0.0326, 0.0346, 0.0349, 0.0350, 0.0355, 0.0379, 0.0384, 0.0394, 0.0394, 0.0412, 0.0419, 0.0425, 0.0461, 0.0464, 0.0468, 0.0471, 0.0495, 0.0501, 0.0521, 0.0571, 0.0588, 0.0597, 0.0628, 0.0679, 0.0685, 0.0715, 0.0766, 0.0780, 0.0942, 0.0960, 0.0988, 0.1223, 0.1343, 0.1781.

**Fig. 3:**
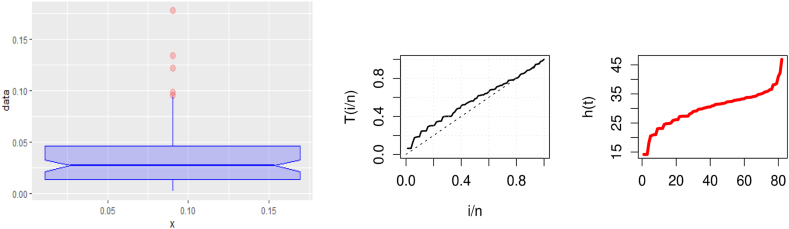
Visualizing survival patterns: Boxplots (left), TTT (center), and Hazard Lines (right) plots for the United Kingdom data set.

This data set has been considered by Ref. [61]. Three plots of the United Kingdom data set's COVID-19 death rates are displayed in Fig. 3: The data set is rising; as indicated by the center TTT plot; the right hazard estimated plot line indicates that the HF is rising, and the left boxplot indicates that there are no outliers in the data.

**Fig. 4:**
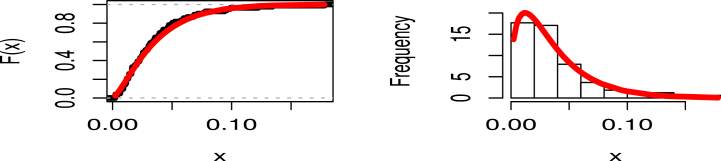
Estimated CDF (left panel) and PDF (right panel) of MK-UEHL distribution with COVID-19 mortality rates of United Kingdom.

Table 5 describes the MLEs of the distribution's parameters and shows the goodness of fit metrics, K**, AIVC, W**, BIVC, HQIVC, CAIVC, and A**. According to Table 5's findings, the MK-UEHL distribution performs better than the UEHL, Kw, Beta, UW, UG, UL, TL, UBXII, and UEP distributions for the provided data. As can be observed, this data set can be modeled fairly well using the UEHL, Kw, Beta, UW, UG, UL, TL, UBXII, and UEP distributions, although the MK-UEHL is the best. Based on a 0.05 significance level, Fig. 4 provides more instances of how the COVID-19 data in the United Kingdom may be fitted through two graphs created using the estimated model parameters. The dataset's histogram is shown with the fitted PDF for the MK-UEHL distribution on the right panel, and the empirical CDF plot with estimated CDF is shown on the left panel. These also guarantee that the data sets fit the MK-UEHL model.

**Table 5:** Assessing model fit for Dataset 1: MLE and SE Evaluation.

Models		Estimates	SE	K**	PV-K**	AIVC	BIVC	CAIVC	HQIVC	W**	A**
MK-UEHL	ς	1.3773	0.1102	0.0514	0.9820	−386.6979	−381.8845	−386.5460	−384.7654	0.0359	0.2706
	κ	32.3407	11.2384								
UEHL	δ	1.2515	0.1030	0.0574	0.9496	−385.0542	−380.2408	−384.9023	−383.1217	0.0555	0.3931
	ϕ	29.3838	9.3197								
Kw	*a*	1.2398	0.1055	0.0597	0.9317	−384.6698	−379.8564	−384.5179	−382.7373	0.0601	0.4227
	b	55.7172	18.2779								
Beta	*a*	1.5114	0.2150	0.0514	0.9819	−386.6620	−381.8485	−386.5101	−384.7294	0.0397	0.2931
	b	40.6947	6.7995								
UW	α	0.0024	0.0003	0.0737	0.7639	−381.6038	−376.7903	−381.4519	−379.6713	0.0988	0.7080
	β	4.3158	0.1099								
UG	α	0.0181	0.0071	0.1081	0.2934	−363.6987	−358.8853	−363.5468	−361.7662	0.2941	1.9827
	β	0.9759	0.0801								
UL	θ	27.1080	2.8942	0.1159	0.2205	−381.5790	−379.1723	−381.5290	−380.6128	0.0484	0.3495
UBXII	α	0.0299	0.0698	0.4473	0.0000	−187.4295	−182.6160	−187.2776	−185.4969	0.0980	0.6633
	β	26.2239	61.2265								
TL	θ	0.3324	0.0367	0.3946	0.0000	−254.3872	−251.9805	−254.3372	−253.4209	0.0677	0.3538
UEP	α	1.1858	0.0963	0.0644	0.8861	−369.4508	−362.2306	−369.1431	−366.5520	0.0714	0.4944
	β	0.0672	0.2055								
	λ	1.8135	6.5793

**Fig. 5:**
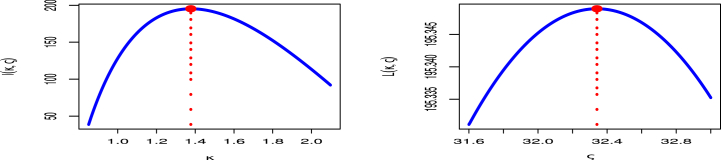
Profile MLE for κ (left panel) and ς (right panel) of MK-UEHL distribution with COVID-19 mortality rates of United Kingdom.

As can be seen from the charts, the COVID-19 mortality rates of the United Kingdom data set behave quite well, with the two roots of the parameters being at their greatest global value. To confirm that the estimates have distinct points, Fig. 5 showes a contour plot of MLE for κ in left and MLE for ς in right and the log-likelihood of the MK-UEHL distribution.

The COVID-19 mortality rates within the United Kingdom dataset are derived from the following PTII-CS schemes:

Sch. 1: Data are “0.0023 0.0023 0.0023 0.0046 0.0065 0.0067 0.0069 0.0069 0.0091 0.0093 0.0093 0.0093 0.0111 0.0115 0.0119 0.0138 0.0159 0.0161 0.0162 0.0162 0.0163 0.0180 0.0187 0.0202 0.0230 0.0230 0.0245 0.0251 0.0255 0.0275 0.0295 0.0297 0.0300 0.0302 0.0326 0.0349 0.0350 0.0355 0.0384 0.0394 0.0394 0.0419 0.0425 0.0461 0.0464 0.0495 0.0501 0.0521 0.0571 0.0588 0.0597 0.0679 0.0715 0.0766 0.0780 0.0942 0.0960 0.0988 0.1343 0.1781”, and *R* is “2 2 2 2 2 2 2 2 2 2 2 0 0 0 0 0 0 0 0 0 0 0 0 0 0 0 0 0 0 0 0 0 0 0 0 0 0 0 0 0 0 0 0 0 0 0 0 0 0 0 0 0 0 0 0 0 0 0 0 0”.

Sch. 2: Data are “0.0023 0.0023 0.0023 0.0046 0.0065 0.0067 0.0069 0.0091 0.0093 0.0093 0.0111 0.0115 0.0116 0.0119 0.0136 0.0138 0.0159 0.0161 0.0162 0.0162 0.0180 0.0187 0.0202 0.0207 0.0225 0.0230 0.0230 0.0239 0.0251 0.0255 0.0255 0.0271 0.0275 0.0300 0.0302 0.0312 0.0314 0.0326 0.0346 0.0355 0.0379 0.0384 0.0394 0.0394 0.0419 0.0425 0.0461 0.0464 0.0471 0.0495 0.0501 0.0521 0.0588 0.0628 0.0685 0.0766 0.0780 0.0960 0.1343 0.1781”, and R is “22 0 0 0 0 0 0 0 0 0 0 0 0 0 0 0 0 0 0 0 0 0 0 0 0 0 0 0 0 0 0 0 0 0 0 0 0 0 0 0 0 0 0 0 0 0 0 0 0 0 0 0 0 0 0 0 0 0 0 0”.

Sch. 3: Data are “0.0023 0.0023 0.0023 0.0046 0.0065 0.0067 0.0069 0.0069 0.0091 0.0093 0.0093 0.0093 0.0111 0.0115 0.0116 0.0116 0.0119 0.0133 0.0136 0.0138 0.0138 0.0159 0.0161 0.0162 0.0162 0.0162 0.0163 0.0180 0.0187 0.0202 0.0207 0.0208 0.0225 0.0230 0.0230 0.0239 0.0245 0.0251 0.0255 0.0255 0.0271 0.0275 0.0295 0.0297 0.0300 0.0302 0.0312 0.0314 0.0326 0.0346 0.0349 0.0350 0.0355 0.0379 0.0384 0.0394 0.0394 0.0412 0.0419 0.0425”, and *R* is “0 0 0 0 0 0 0 0 0 0 0 0 0 0 0 0 0 0 0 0 0 0 0 0 0 0 0 0 0 0 0 0 0 0 0 0 0 0 0 0 0 0 0 0 0 0 0 0 0 0 0 0 0 0 0 0 0 0 0 22”.

**Fig. 6:**
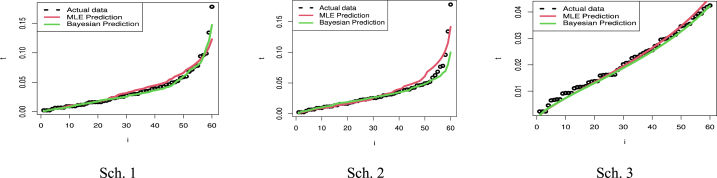
Comparison between actual, MLE, and Bayesian prediction observations based on Scheme 1 (left panel), Scheme 2 (center panel) and Scheme 3 (right panel): United Kingdom data set.

**Fig. 7:**
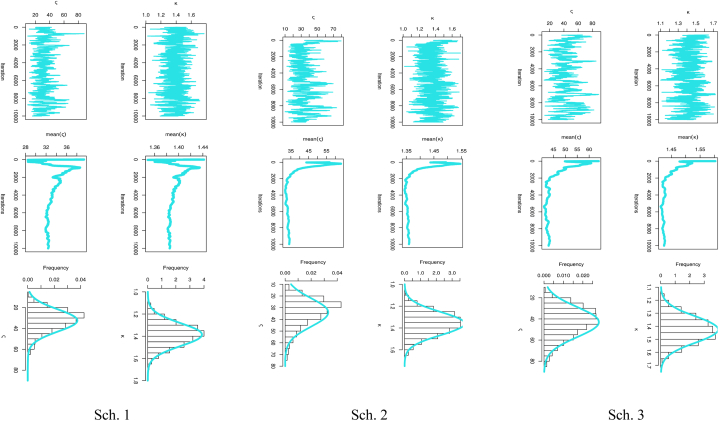
MCMC iteration checked by trace (top), convergence analysis (center), and normality (bottom) plots for parameters MK-UEHL based on the PTII-CS: United Kingdom data set.

Fig. 6 presents the predictive points for the kth order statistic based on two sample predictions of COVID-19 mortality rates in the United Kingdom. The left panel illustrates the results for Sch. 1, the center panel for Sch. 2, and the right panel for Sch. 3. It can be seen from Table 6 and Fig. 6 how closely the outcomes of the MLE and Bayesian approaches to value prediction match the actual value. Also, Table 6 discusses the estimation of parameters based on PTII-CS with different schemes. As well, Sch. 2 for MLE results has the smallest SE, while Sch. 1 for BEs for parameters of the MK-UEHL based on the PTII-CS of the United Kingdom data set has the smallest SE. As illustrated in Fig. 7, the MCMC findings reveal symmetric posterior density histograms, trace (top), normality (bottom), and convergence (center) measures for parameters MK-UEHL based on PTII-CS of the first data set for each scheme under this study.

**Table 6:** MLE, Bayesian estimation for parameters MK-UEHL based on PTII-CS: United Kingdom data set.

			ML		Bayesian		Prediction			
*m*	Scheme		Estimates	SE	Estimates	SE	k	Actual	ML	Bayesian
60	1	ς	1.3930	0.1299	1.3850	0.1035	40	0.0394	0.0435	0.0414
		κ	32.2513	13.4403	32.3980	10.6414	50	0.0588	0.0636	0.0624
	2	ς	1.3569	0.1180	1.3596	0.1051	40	0.0355	0.0399	0.0347
		κ	32.3531	12.3877	34.0285	12.0483	50	0.0495	0.0611	0.0486
	3	ς	1.3666	0.1215	1.4249	0.1006	40	0.0255	0.0264	0.0251
		κ	32.3800	13.8421	42.9672	13.1404	50	0.0346	0.0347	0.0346

Dataset II presents the daily COVID-19 mortality rates in Saudi Arabia from July 22, 2021, to August 26, 2021. The details are as follows: 0.2375, 0.2962, 0.2167, 0.2752, 0.2353, 0.2347, 0.1951, 0.2140, 0.2329, 0.2711, 0.2126, 0.2314, 0.1924, 0.2113, 0.2683, 0.2487, 0.2674, 0.1716, 0.2666, 0.2091, 0.2278, 0.1706, 0.2271, 0.1890, 0.2077, 0.2452, 0.1319, 0.2259, 0.1504, 0.1879, 0.1689, 0.2063, 0.2249, 0.1686, 0.1310, 0.1497.

**Fig. 8:**
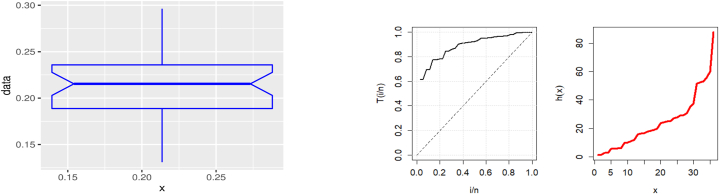
Visualizing survival patterns: Boxplots (left), TTT (center), and Hazard Lines (right) plots for Saudi Arabia.

Based on this data, Fig. 8 provides three plots, including boxplot (left), TTT (center), and hazard rate (right), respectively. The first plot explains that there are no outliers; the center plot shows that the data set is rising; and the last plot shows that the hazard rate is rising.

**Fig. 9:**
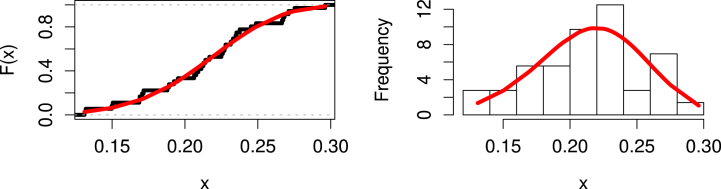
Estimated CDF (left) and PDF (right) of MK-UEHL distribution with COVID-19 mortality rates in Saudi Arabia.

Table 7 describes the MLEs of the distribution's parameters and shows the goodness of fit metrics, K**, AIVC, W**, CAIVC, HQIVC, BIVC, and A**. According to Table 7's findings, the MK-UEHL distribution performs better than the UEHL, Kw, Beta, UW, UG, UL, TL, UBXII, UGLBXII, and UEP distributions for the second data set. As can be observed, the provided data can be modeled fairly well using each of the UEHL, Kw, Beta, UW, UGLBXII, UG, UL, TL, UBXII, and UEP distributions, although the MK-UEHL is the best. Two graphs that were produced using the estimated model parameters are shown in Fig. 9 as additional examples of how the suggested COVID-19 data can be fitted. The dataset's histogram is shown with the fitted PDF for the MK-UEHL distribution on the right, and the empirical CDF plot with estimated CDF is shown on the left. These also guarantee that the data sets fit the MK-UEHL model.

**Table 7:** Assessing model fit for Dataset 2: MLE and SE Evaluation.

Models		Estimates	SE	K**	PV-K**	AIVC	BIVC	CAIVC	HQIVC	W**	A**
MK-UEHL	ς	6.6287	0.7973	0.0760	0.9753	−125.2762	−122.1092	−124.9126	−124.1709	0.0329	0.2349
	κ	6178.5994	31.4155								
UEHL	δ	6.0655	0.7697	0.0792	0.9641	−125.1855	−122.0184	−124.8218	−124.0801	0.0330	0.2393
	ϕ	3670.3422	388.2132								
Kw	*a*	6.0645	0.7584	0.0793	0.9638	−125.1855	−122.0185	−124.8219	−124.0801	0.0330	0.2392
	b	7328.1179	744.1120								
Beta	*a*	20.8638	4.8800	0.1126	0.7091	−123.7901	−120.6230	−123.4264	−122.6847	0.0636	0.3992
	b	76.6960	18.0965								
UW	α	0.0203	0.0110	0.1633	0.2624	−116.2105	−113.0435	−115.8469	−115.1051	0.1824	1.0950
	β	7.7557	0.9132								
UG	α	0.0022	0.0004	0.2073	0.0780	−108.4628	−105.2958	−108.0992	−107.3574	0.2202	1.3174
	β	3.7155	0.1302								
UL	θ	4.3132	0.6208	0.4142	0.0000	−54.9480	−53.3645	−54.8304	−54.3953	0.0542	0.3456
UBXII	α	0.0298	0.1042	0.4136	0.0000	−64.4153	−61.2482	−64.0516	−63.3099	0.0336	0.2376
	β	76.6443	68.1812								
TL	θ	1.0187	0.1698	0.5041	0.0000	−30.4937	−28.9102	−30.3760	−29.9410	0.0665	0.4155
UGLBXII	α	0.5473	0.3169	0.0797	0.9625	−121.8929	−117.1424	−121.1429	−120.2349	0.0338	0.2439
	β	9.0927	2.9089								
	λ	1.4516	0.0760								
UEP	ς	4.6961	0.6030	0.0876	0.9226	−88.0391	−83.2885	−87.2891	−86.3810	0.0361	0.2617
	κ	0.4106	2.1538								
	δ	4.2896	105.6766

**Fig. 10:**
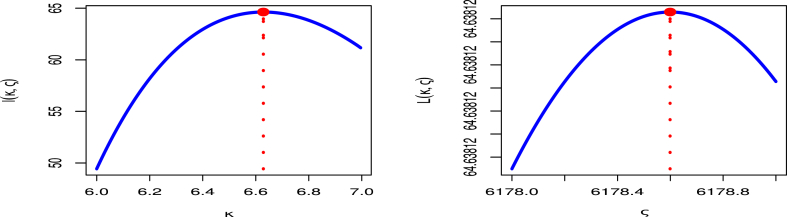
Profile MLE for κ (left panel) and ς (right panel) of MK-UEHL distribution with COVID-19 mortality rates in Saudi Arabia.

From Fig. 10, it's evident that the dataset exhibit a smooth trend, with the parameters' two roots reaching their highest values globally. To ensure that these estimates have unique points, Fig. 10 confirms the uniqueness of the MLE for κ in left panel and MLE for ς in right panel by demonstrating that it is the only value that maximizes the log-likelihood function.

The Saudi Arabia dataset's COVID-19 mortality rates were determined using PTII-CS methodologies.

Sch. 1: Data are “0.1310 0.1319 0.1497 0.1504 0.1686 0.1706 0.1716 0.1879 0.1890 0.1924 0.1951 0.2077 0.2091 0.2140 0.2167 0.2249 0.2259 0.2278 0.2314 0.2329 0.2347 0.2353 0.2375 0.2452 0.2487 0.2666 0.2683 0.2711 0.2752 0.2962”, *R* is “2 2 2 0 0 0 0 0 0 0 0 0 0 0 0 0 0 0 0 0 0 0 0 0 0 0 0 0 0 0”.

Sch. 2: Data are“0.1310 0.1319 0.1497 0.1686 0.1689 0.1706 0.1716 0.1879 0.1890 0.1951 0.2063 0.2077 0.2126 0.2140 0.2249 0.2259 0.2271 0.2278 0.2314 0.2329 0.2347 0.2353 0.2375 0.2452 0.2487 0.2666 0.2683 0.2711 0.2752 0.2962”, and *R* is “6 0 0 0 0 0 0 0 0 0 0 0 0 0 0 0 0 0 0 0 0 0 0 0 0 0 0 0 0 0”.

Sch. 3: Data are “0.1310 0.1319 0.1497 0.1504 0.1686 0.1689 0.1706 0.1716 0.1879 0.1890 0.1924 0.1951 0.2063 0.2077 0.2091 0.2113 0.2126 0.2140 0.2167 0.2249 0.2259 0.2271 0.2278 0.2314 0.2329 0.2347 0.2353 0.2375 0.2452 0.2487”, and *R* is “0 0 0 0 0 0 0 0 0 0 0 0 0 0 0 0 0 0 0 0 0 0 0 0 0 0 0 0 0 6”.

**Fig. 11:**
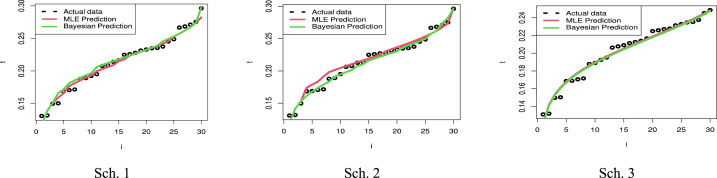
Comparison between actual, MLE, and Bayesian prediction observations based on Scheme 1 (left panel), Scheme 2 (center panel) and Scheme 3 (right panel): Saudi Arabia data set.

Fig. 11 presents predictive points for the k-th order statistic in a two-sample prediction scenario, using the observed sample data for COVID-19 mortality rates in Saudi Arabia. The left panel illustrates the results for Scheme 1, the center panel for Scheme 2, and the right panel for Scheme 3. By examining Table 8 and Fig. 11, it becomes apparent how closely the results of both ML and Bayesian estimation methods align with the actual values. Table 8 also delves into parameter estimation using PTII-CS with different approaches. Notably, scheme 1 for both MLE and BE results exhibits the smallest SE.

**Table 8:** MLE, Bayesian estimation for parameters MK-UEHL based on PTII-CS: Saudi Arabia data set.

			ML		Bayesian		prediction			
*m*	Sch.		Estimates	SE	Estimates	SE	k	Actual	ML	Bayesian
30	1	ς	6.4672	0.8975	6.4705	0.2120	20	0.2329	0.2325	0.2326
		κ	4757.4508	611.5916	4758.0236	26.9996	25	0.2487	0.2529	0.2520
	2	ς	6.6471	0.9663	6.6461	0.2137	20	0.2329	0.2364	0.2305
		κ	6013.2418	830.9563	6011.8531	38.2085	25	0.2487	0.2534	0.2496
	3	ς	6.9352	1.2938	6.9168	0.1894	20	0.2249	0.2188	0.2179
		κ	10249.9026	977.8709	10249.7304	87.4632	25	0.2329	0.2325	0.2316

**Fig. 12:**
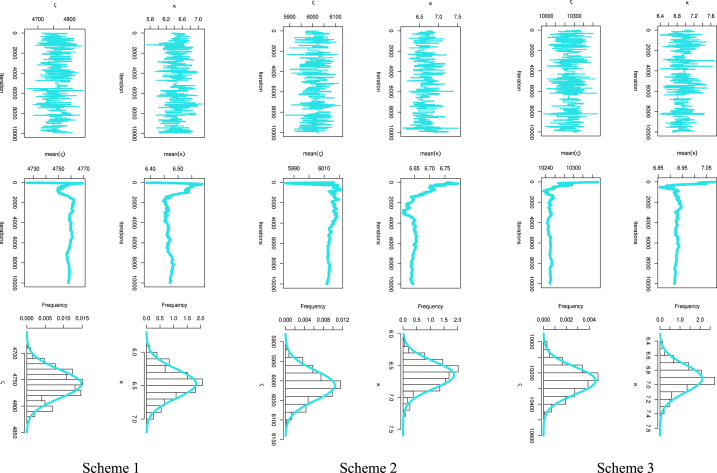
MCMC iteration checked by trace (top), convergence analysis (center), and normality (bottom) plots for parameters MK-UEHL based on the PTII-CS: Saudi Arabia data set.

In Fig. 12, the findings from MCMC analysis depict symmetric posterior density histograms, trace (top), normality (bottom), and convergence (center) measures for the MK-UEHL parameters, specifically those derived from PTII-CS in the Saudi Arabia dataset for each scheme explored in this study.

## 8 Summary and conclusion

This study presents the novel KM-UEHL distribution, an improved variant of the unit exponentiated half logistic model. Effective modeling of data on the unit interval is possible with the new KM-UEHL distribution. The efficacy of the KM-UEHL distribution in reproducing a wide range of data is demonstrated by its versatility with respect to both density and hazard rate. Its key characteristics are carefully examined, including moments analytical expression, quantile function, incomplete moments, residual moments, entropy measurements, and stress-strength reliability. A progressive Type-II censored method is utilized to predict future data using the KM- UEHL distribution. The maximum likelihood, Bayesian (point and interval), is generated for future order statistics using a two-sample prediction. The MCMC samples generated by the M − H technique with Gibbs sampling are used since it is challenging to reduce the expected posterior density function. Furthermore, it offers the upper- order statistics' prediction interval. The theoretical conclusions are explained through a numerical analysis, and their potential applications are illustrated using actual COVID-19 data sets. Bayesian estimation performs better on real data sets when compared to the ML technique since the prediction observations are nearly to actual values. Further research might examine the statistical inference of SS reliability using some scenario for the KM-UEHL distribution [[62], [63], [64]]. The proposed model can be extended to analyze data with complex censoring patterns, beyond the basic schemes currently considered [65,66]. Further, the model's ability to classify different types of genetic mutations can be examined [67,68].

## Data availability

All data existing in the paper are associated with its references and sources.

## Funding

Funding:This work was supported and funded by the Deanship of Scientifc Research at Imam Mohammad Ibn Saud Islamic University (IMSIU) (grant number IMSIU-RG23142).

## CRediT authorship contribution statement

**Naif Alotaibi:** Writing – review & editing, Methodology, Investigation, Funding acquisition, Formal analysis. **A.S. Al-Moisheer:** Resources, Investigation, Formal analysis, Data curation. **Amal S. Hassan:** Writing – review & editing, Writing – original draft, Software, Resources, Methodology, Data curation. **Ibrahim Elbatal:** Writing – original draft, Resources, Methodology, Formal analysis, Data curation. **Salem A. Alyami:** Writing – review & editing, Methodology, Formal analysis, Data curation. **Ehab M. Almetwally:** Writing – original draft, Software, Resources, Methodology.

## Declaration of competing interest

The authors declare that they have no known competing financial interests or personal relationships that could have appeared to influence the work reported in this paper.

## Appendix 1

$$\frac{\partial^{2}l^{*}\left(\varpi \right)}{\partial \varsigma^{2}}=\frac{-m}{\varsigma^{2}}+\sum_{i=1}^{m}H_{i}\left(t_{i},\kappa ,\varsigma \right){\left[\ln\;D_{i}\left(t,\kappa \right)\right]}^{2}+\sum_{i=1}^{m}\frac{\left(e-1\right)R_{i}e^{-\left(1-H_{i}\left(t_{i},\kappa ,\varsigma \right)\right)}H_{i}\left(t_{i},\kappa ,\varsigma \right){\left[\ln\left(D_{i}\left(t,\kappa \right)\right)\right]}^{2}\left[1+H_{i}\left(t_{i},\kappa ,\varsigma \right)\right]}{\left(e-1\right)-e\left[1-e^{-\left(1-H_{i}\left(t_{i},\kappa ,\varsigma \right)\right)}\right]}+\sum_{i=1}^{m}\frac{\left(e-1\right)R_{i}e^{-\left(1-H_{i}\left(t_{i},\kappa ,\varsigma \right)\right)}H_{i}^{2}\left(t_{i},\kappa ,\varsigma \right){\left[\ln\left(D_{i}\left(t,\kappa \right)\right)\right]}^{2}}{{\left\{\left(e-1\right)-e\left[1-e^{-\left(1-{Η}_{i}\left(t_{i},\kappa ,\varsigma \right)\right)}\right]\right\}}^{2}}$$

$$\begin{array}{l} \frac{\partial^{2}l^{*}\left(\varpi \right)}{\partial \kappa^{2}}=\frac{-m}{\kappa^{2}}-\sum_{i=1}^{m}\frac{\left(\varsigma -1\right)t^{-\kappa }{\left(\ln\;t_{i}\right)}^{2}}{{\left[t_{i}^{-\kappa }-1\right]}^{2}}-\sum_{i=1}^{m}\frac{\left(\varsigma +1\right)t^{-\kappa }{\left(\ln\;t_{i}\right)}^{2}}{\left[1+t_{i}^{-\kappa }\right]}-\sum_{i=1}^{m}\frac{2\varsigma {\left(\ln\;t_{i}\right)}^{2}t_{i}^{\kappa }{\left[D_{i}\left(t,\kappa \right)\right]}^{\varsigma -2}\left[1-t_{i}^{2\kappa }-2t_{i}^{\kappa }\left(\varsigma -1\right)\right]}{{\left(1+t_{i}^{\kappa }\right)}^{4}}+\sum_{i=1}^{m}\frac{2\varsigma {\left(\ln\;t_{i}\right)}^{2}t_{i}^{2\kappa }{\left[D_{i}\left(t,\kappa \right)\right]}^{\varsigma -1}}{{\left(1+t_{i}^{\kappa }\right)}^{4}}\;-\;\sum_{i=1}^{m}\frac{R_{i}e^{*}e^{-\left(1-{Η}_{i}\left(t_{i},\kappa ,\varsigma \right)\right)}\varsigma 2t_{i}^{\kappa }{\left(\ln\;t_{i}\right)}^{2}{Η}_{i}\left(t_{i},\kappa ,\varsigma \right)\left[1-t_{i}^{\kappa }{\left(1-t_{i}^{2\kappa }\right)}^{-1}\left(2{Η}_{i}\left(t_{i},\kappa ,\varsigma \right)+\varsigma \right)\right]}{{\left[\left\{1-e^{*}\left[1-e^{-\left(1-{Η}_{i}\left(t_{i},\kappa ,\varsigma \right)\right)}\right]\right\}\left(1-t_{i}^{2\kappa }\right)\right]}^{2}}-\;\sum_{i=1}^{m}\frac{R_{i}e^{*}e^{-\left(1-{Η}_{i}\left(t_{i},\kappa ,\varsigma \right)\right)}\varsigma 2t_{i}^{\kappa }\;\ln\;t_{i}{Η}_{i}\left(t_{i},\kappa ,\varsigma \right)\left[2t_{i}^{\kappa }\;\ln\;t_{i}\left(1-e^{*}\left[1-e^{-\left(1-{Η}_{i}\left(t_{i},\kappa ,\varsigma \right)\right)}\right]\right)+2t_{i}^{\kappa }\varsigma \;\ln\;t_{i}{Η}_{i}\left(t_{i},\kappa ,\varsigma \right)\right]}{{\left[\left\{1-e^{*}\left[1-e^{-\left(1-{Η}_{i}\left(t_{i},\kappa ,\varsigma \right)\right)}\right]\right\}\left(1-t_{i}^{2\kappa }\right)\right]}^{2}} \end{array}$$

$$\frac{\partial l^{*}\left(\varpi \right)}{\partial \kappa \partial \varsigma }=\sum_{i=1}^{m}\frac{\ln\;t_{i}}{\left[t_{i}^{-\kappa }-1\right]}-\sum_{i=1}^{m}\frac{\ln\;t_{i}}{\left[1+t_{i}^{-\kappa }\right]}-\;\sum_{i=1}^{m}\frac{R_{i}e^{*}e^{-\left(1-{Η}_{i}\left(t_{i},\kappa ,\varsigma \right)\right)}\varsigma 2t_{i}^{\kappa }\;\ln\;t_{i}{Η}_{i}\left(t_{i},\kappa ,\varsigma \right)\left[1+{Η}_{i}\left(t_{i},\kappa ,\varsigma \right)\right]\ln\left[D_{i}\left(t,\kappa \right)\right]}{\left\{1-e^{*}\left[1-e^{-\left(1-{Η}_{i}\left(t_{i},\kappa ,\varsigma \right)\right)}\right]\right\}\left(1-t_{i}^{2\kappa }\right)}-\;\sum_{i=1}^{m}\frac{2t_{i}^{\kappa }\;\ln\;t_{i}{\left[D_{i}\left(t,\kappa \right)\right]}^{\varsigma -1}\left[1+\varsigma \left[D_{i}\left(t,\kappa \right)\right]\right]}{{\left(1+t_{i}^{\kappa }\right)}^{2}}-\;\sum_{i=1}^{m}\frac{R_{i}{e^{*}}^{2}e^{-2\left(1-{Η}_{i}\left(t_{i},\kappa ,\varsigma \right)\right)}\varsigma 2t_{i}^{\kappa }\;\ln\;t_{i}{Η}_{i}^{2}\left(t_{i},\kappa ,\varsigma \right)\ln\left[D_{i}\left(t,\kappa \right)\right]}{\left(1-t_{i}^{2\kappa }\right){\left[\left\{1-e^{*}\left[1-e^{-\left(1-{Η}_{i}\left(t_{i},\kappa ,\varsigma \right)\right)}\right]\right\}\right]}^{2}}\text{,}$$
